# Sal-type ABC-F proteins: intrinsic and common mediators of pleuromutilin resistance by target protection in staphylococci

**DOI:** 10.1093/nar/gkac058

**Published:** 2022-02-07

**Authors:** Merianne Mohamad, David Nicholson, Chayan Kumar Saha, Vasili Hauryliuk, Thomas A Edwards, Gemma C Atkinson, Neil A Ranson, Alex J O’Neill

**Affiliations:** Astbury Centre for Structural Molecular Biology and School of Molecular & Cellular Biology, Faculty of Biological Sciences, University of Leeds, Leeds, UK; Astbury Centre for Structural Molecular Biology and School of Molecular & Cellular Biology, Faculty of Biological Sciences, University of Leeds, Leeds, UK; Department of Molecular Biology, Umeå University, 90187 Umeå, Sweden; Department of Experimental Medical Science, Lund University, 221 00 Lund, Sweden; Department of Molecular Biology, Umeå University, 90187 Umeå, Sweden; Department of Experimental Medical Science, Lund University, 221 00 Lund, Sweden; Astbury Centre for Structural Molecular Biology and School of Molecular & Cellular Biology, Faculty of Biological Sciences, University of Leeds, Leeds, UK; Department of Molecular Biology, Umeå University, 90187 Umeå, Sweden; Department of Experimental Medical Science, Lund University, 221 00 Lund, Sweden; Astbury Centre for Structural Molecular Biology and School of Molecular & Cellular Biology, Faculty of Biological Sciences, University of Leeds, Leeds, UK; Astbury Centre for Structural Molecular Biology and School of Molecular & Cellular Biology, Faculty of Biological Sciences, University of Leeds, Leeds, UK

## Abstract

The first member of the pleuromutilin (PLM) class suitable for systemic antibacterial chemotherapy in humans recently entered clinical use, underscoring the need to better understand mechanisms of PLM resistance in disease-causing bacterial genera. Of the proteins reported to mediate PLM resistance in staphylococci, the least-well studied to date is Sal(A), a putative ABC-F NTPase that—by analogy to other proteins of this type—may act to protect the ribosome from PLMs. Here, we establish the importance of Sal proteins as a common source of PLM resistance across multiple species of staphylococci. Sal(A) is revealed as but one member of a larger group of Sal-type ABC-F proteins that vary considerably in their ability to mediate resistance to PLMs and other antibiotics. We find that specific *sal* genes are intrinsic to particular staphylococcal species, and show that this gene family is likely ancestral to the genus Staphylococcus. Finally, we solve the cryo-EM structure of a representative Sal-type protein (Sal(B)) in complex with the staphylococcal 70S ribosome, revealing that Sal-type proteins bind into the E site to mediate target protection, likely by displacing PLMs and other antibiotics via an allosteric mechanism.

## INTRODUCTION

Pleuromutilin (PLM) antibiotics inhibit bacterial protein synthesis by binding to the large ribosomal subunit at the peptidyltransferase centre (PTC) and preventing the requisite positioning of the A- and P-site tRNA for peptide bond formation (1). The PLM class has a long history (>40 years) of use in veterinary medicine for the prevention and treatment of bacterial infection. Since 2007, PLMs have also been in human use in the form of retapamulin, which is approved for topical application to treat superficial infections caused by *Staphylococcus aureus* and other Gram-positive pathogens (2). In 2019, lefamulin became the first systemic PLM to be approved in humans, administered either intravenously or orally for the treatment of community-acquired bacterial pneumonia (3).

Growing use of this antibiotic class in human medicine underscores the need for a more complete understanding of the nature of PLM resistance. At present, resistance to PLMs appears to be relatively uncommon amongst the major target genera against which retapamulin and lefamulin are deployed, such as the staphylococci; prevalence studies in *S. aureus* and non-aureus staphylococci report rates for resistance of 0.1–2.6% (4–6). Nevertheless, several PLM resistance determinants have been identified in this genus. For example, the *cfr* gene confers resistance through methylation of 23S ribosomal RNA, a modification that serves to protect the ribosome from PLM and several other antibiotic classes whose binding sites lie in close proximity (phenicols, lincosamides, group A streptogramins, and oxazolidinones) (7). PLM resistance can also result from mutational change in the ribosome, involving amino acid substitutions in ribosomal proteins L3 and L4, or nucleotide substitutions in 23S rRNA (8,9). However, the above resistance mechanisms are not widespread in staphylococci, and do not therefore represent common causes of clinically-significant PLM resistance at present (6,10).

Of greater importance for PLM resistance in staphylococci—especially in view of their greater collective prevalence—are several antibiotic resistance (ARE) ABC-F proteins, exemplified by the Vga- and Lsa groups (11). In contrast to the more canonical members of the ATP-Binding Cassette (ABC) superfamily that participate in drug resistance, ABC-F proteins such as these do not transport antibiotic across membranes. Instead, the Vga- and Lsa-type proteins mediate resistance to PLMs and other protein synthesis inhibitors by target protection; they bind to the ribosome to drive antibiotic release (12,13), although the precise mechanism by which the latter occurs remains to be clarified. Of these two groups, the *vga*-type genes currently appear to represent the major PLM-resistance determinants in *S. aureus* and some non-aureus staphylococci (6,14), and are typically associated with mobile genetic elements such as plasmids that facilitate their spread (15). Of the *lsa*-type genes, *lsa(E)* was the first gene to be characterised in staphylococci, and several other variants of this determinant have subsequently been identified in human and veterinary *S. aureus* isolates, again typically in association with plasmids (16). A further ARE ABC-F member known to mediate PLM resistance in staphylococci is a relatively poorly-characterized protein known as Sal(A) (17), which belongs to the subfamily of ABC-Fs designated ARE6 (18). The *sal(A)* gene was first identified as a cause of resistance to lincosamides and group A streptogramins in *Staphylococcus sciuri* (17), and only later shown to be involved in PLM resistance (19). By analogy to the Vga- and Lsa-type proteins, Sal proteins may physically associate with the ribosome to protect it from antibiotics, though this has to date not been demonstrated.

Here, we establish the importance of *sal*-type genes as a common source of intrinsic PLM resistance across multiple species of staphylococci. Sal(A) is revealed as but one member of a larger group of Sal-type ABC-F proteins that is likely ancestral to the genus, and which shows considerable variation in the ability to mediate resistance to antibiotics. We solve the cryo-EM structure of a Sal-type protein in complex with the staphylococcal ribosome, confirming that Sal-type proteins do indeed mediate target protection, likely by displacing PLMs and other antibiotics from the ribosome via an allosteric mechanism.

## MATERIALS AND METHODS

### Bacteria, culture conditions and susceptibility testing

The collection of non-aureus staphylococci used in this study (*n* = 363) comprised 214 human isolates recovered from hospitals in the UK, Canada and Italy between 2012 and 2016 and 149 veterinary isolates obtained from the Royal Veterinary College (London, UK). Bacteria were routinely cultured at 37°C using cation-adjusted Mueller Hinton agar (MHA) or broth (MHB) (Sigma-Aldrich) for 18–24 h. To detect PLM resistance, bacteria (10^4^ CFU) were spotted onto MHA containing retapamulin (AdooQ BioScience) at 2 μg/ml (20). Strains that grew on these plates were subjected to susceptibility determinations with retapamulin and other PLMs (tiamulin [Sigma-Aldrich] and lefamulin [DC Chemicals]) by broth microdilution according to CLSI methodology (21). PCR amplification and DNA sequencing of the 16S rDNA (22) or the *rpoB* gene (23) were employed for species identification of resistant isolates.

### Determining the genetic basis for PLM resistance

Retapamulin-resistant isolates were screened for the presence of known staphylococcal PLM-resistance determinants by PCR using GoTaq PCR mastermix (Promega) and oligonucleotide primers (Eurofins Genomics) designed to generate amplicons from *vga-, lsa-*, and *sal-*type genes (Supplementary Table S1). DNA sequencing of the resulting amplicons was performed (i) to confirm that they correspond to the resistance gene in question and (ii) to detect sequence variants.

Where appropriate, strains were subjected to whole genome sequencing (WGS). Genomic DNA was isolated using the PurElute™ Bacterial Genomic Purification Kit (Edge BioSystems) essentially according to the manufacturer's instructions, though bacteria were resuspended in the first instance in spheroblast buffer containing lysostaphin (100 μg/ml) and incubated at 37°C for 45 min. WGS was performed on the Illumina platform at the Next Generation Sequencing Facility (St. James's Hospital, University of Leeds) or at MicrobesNG (www.microbesng.uk), and DNA sequence data assembled using CLC Genomic workbench (CLC Bio) and annotated using RAST (www.rast.theseed.org).

### Confirmation and characterization of PLM resistance genes

Putative PLM resistance genes identified in this study were introduced into a PLM-susceptible *S. aureus* host to assess their ability to confer resistance. DNA fragments corresponding to these genes were either generated by PCR-amplification with Phusion® High-Fidelity DNA Polymerase (NEB) using oligonucleotide primers described in Supplementary Table S1 or were obtained by synthesis (Genewiz). PCR amplicons and synthesized DNA fragments were digested with KpnI and EcoRI (NEB) to enable directional ligation into similarly-digested expression plasmid pRMC2 (24) for transformation of *Escherichia coli* XL10-Gold (Agilent Technologies). DNA-sequence verified constructs were then introduced into *S. aureus* RN4220 (25) by electroporation (12). Transformants were grown in cation-adjusted MHB at 37°C with vigorous aeration to an OD_625_ of 0.6, and expression induced with anhydrotetracycline hydrochloride (ATc) (Sigma-Aldrich) at a final concentration of 100 ng/ml for 3 h. Susceptibility testing of these induced cultures was carried out as above, using MHB supplemented with ATc (100 ng/ml).

### Sequence alignment, phylogenetic analysis and gene neighbourhood analysis

Staphylococcal sequences in the ARE6 (Sal) subfamily were extracted from an existing database of ABC-F proteins (18). Additional Sal and cysteine desulfurase (gene immediately downstream of *sal(A)*) sequences were identified in complete staphylococcal genomes deposited in NCBI using, respectively, HMMR (26) (in the strategy described in (18)), and Blastp with an *E* value cut-off of 1e^–100^ (27). Other ARE ABC-F proteins were retrieved from the CARD database (28). Sequences were aligned using MAFFT version v6.861b with default settings (29). Maximum Likelihood phylogenetic analysis was carried out with RaxML version 8.2.12 (30) on the Cipres Science Gateway (31) with the LG model of substitution and 100 bootstrap replicates. Alignment positions with >50% gaps were removed, as well as the ambiguously aligned C-terminal domain, prior to phylogenetic analysis. For gene neighbourhood analysis, FlaGs (Flanking Genes) (32) was run with default settings, with six flanking genes either side of the query gene encoding either Sal or cysteine desulfurase.

### Generation and purification of Sal(B)•ribosome complexes

A DNA fragment encoding the EQ_2_ mutant of the Sal(B) protein fused with a C-terminal FLAG_3_ tag was obtained by synthesis (Genewiz), and introduced into *S. aureus* SH1000 (33,34) on plasmid pRMC2, essentially as described above. A 400 ml culture of this strain was grown at 37°C in LB media supplemented with 20 μg/ml chloramphenicol to an OD_600_ of ∼0.5, before inducing expression of the protein with 100 ng/ml ATc for 60 min. Bacteria were harvested by centrifugation and the resulting cell pellet resuspended in 2 ml HEPES:Polymix buffer (20 mM HEPES–KOH pH 7.5, 5 mM Mg(OAc)_2_, 95 mM KCl, 5 mM NH_4_Cl, 0.5 mM CaCl_2_, 8 mM putrescine, 1 mM spermidine) (35) supplemented with 0.5 mM ATP, 2 mM DTT and 1 tablet of complete protease inhibitor cocktail (Roche). The resuspended cells were lysed using silica beads (Lysing Matrix B; MP Biomedicals) on a FastPrep-24 Classic homogenizer (MP Biomedicals), with 4 cycles of 20 s at 6 m/s with 60 seconds of cooling on ice between cycles. Beads and cell debris were removed by centrifugation at 30 000 × *g* for 30 min.

The clarified lysate was incubated with 200 μl of HEPES:Polymix buffer-washed anti-FLAG M2 affinity gel (Sigma-Aldrich) for 2 h at 4°C, with periodic, gentle mixing. Beads were washed with 500 μl HEPES:Polymix buffer four times, and the bound material recovered following incubation in the presence of 300 μl of 0.1 mg/ml 3× FLAG peptide solution (Sigma-Aldrich) prepared in HEPES:Polymix buffer for 20 minutes at 4°C (12).

### Cryo-EM structure determination of the Sal(B)•ribosome complex

A Quantifoil grid (R1.2/1.3, 400 Cu mesh, with a 2 nm carbon overlay) was glow discharged (Quorum GloQube; 10 mA, 30 s) and then transferred to the humidity- and temperature-controlled chamber of a Vitrobot Mark IV (Thermo Fisher Scientific; 100% humidity, 4°C). An aliquot (3 μl) of the Sal(B)•ribosome elution fraction was applied to the grid, excess sample immediately removed by blotting, and vitrification performed by plunging the grid into liquid nitrogen-cooled liquid ethane.

Data were collected on a Thermo Fisher Scientific Titan Krios electron microscope (Astbury Biostructure Laboratory, University of Leeds) at 300 kV. The sample was exposed to an electron dose of 60 e^–^/Å^2^ across 8.0 s. 847 micrograph movies were recorded by a Gatan Bioquantum-K2 detector in counting mode, split into frames which each received a dose of 1.20 e^–^/Å^2^. A nominal magnification of 130 000× was applied, resulting in a final object sampling of 1.07 Å/pixel. A defocus range of −0.8 to −2.6 μm was used.

The cryo-EM image processing pipeline is summarised in Supplementary Figure S2. Drift-corrected and dose-corrected averages of each movie were created using MOTIONCOR2 (36), and the contrast transfer functions estimated using Gctf (37). All subsequent image processing steps were carried out using RELION 3.1 (38). 99 615 particles were picked using Laplacian-of-Gaussian autopicking, which were extracted with 4× binning. Reference-free 2D classification was used to prune this dataset by removing particles contributing to lowly populated classes lacking high-resolution features. The remaining 67,391 particles were re-extracted without binning and subjected to 3D classification to remove further junk particles, leaving 67 139 particles that were aligned and refined in 3D using a 60 Å low-pass filtered 3D class as a starting model. Rounds of Bayesian polishing and CTF refinement were performed until the resolution of the map stopped improving. 3D classification without particle alignment was performed to remove further poorly-aligned particles, leaving 64, 101 particles. Focussed 3D classification was then performed using a mask around the E and P sites of the ribosome to yield classes containing E- and P-site density. 59 889 particles were assigned to these classes, which were aligned and refined in 3D, yielding a reconstruction with a global resolution of 2.9 Å after solvent masking (Supplementary Figure S3A, C). Multibody refinement was performed using soft extended masks to define the 50S, 30S body and 30S head as rigid bodies, yielding reconstructions for the 50S, 30S body and 30S head at estimated resolutions of 2.8, 3.0 and 3.0 Å respectively (Supplementary Figure S3B, D–F). Final resolutions were estimated using the gold-standard Fourier shell correlation (FSC = 0.143) criterion.

The sharpened reconstructions were low-pass filtered according to local resolution, estimated using RELION’s own implementation. These maps were used to make figures containing maps coloured by local resolution and for model building and refinement. Specifically, the consensus map was used to build models for the 50S subunit rRNA and ribosomal proteins, Sal(B) and the P-site tRNA, and the 30S body and 30S head multibody maps used to build models for the 30S subunit rRNA and ribosomal proteins.

### Atomic model building of the Sal(B)•ribosome complex

The cryo-EM structure of the *S. aureus* ribosome (PDB 6S0X) (39) was used as a starting model for the ribosomal proteins and rRNAs, *E. coli* P-site initiator tRNA_i_^fMet^ (PDB 5MDZ) (40) as a starting model for the distorted P-site tRNA, and a homology model was generated for EQ_2_-Sal(B) using the SWISS model server (41). These were rigid-body fitted into the cryo-EM reconstructions using UCSF Chimera (42), and the P-site tRNA was mutated to *S. aureus* tRNA_i_^fMet^. A short mRNA was built *de novo* at the P-site codon. COOT (43) was used to manually adjust the models to improve map fit and fix rotamer and Ramachandran outliers, before iterative rounds of model refinement and manual model editing were carried out using PHENIX real space refine (44) and COOT, respectively. Note that the model of the whole ribosome was kept intact, and the 50S, 30S body and 30S head regions were each refined into the appropriate consensus or multibody reconstruction whilst keeping the rest of the model fixed. Regions where the protein or rRNA backbone could not be traced were deleted. The model was validated using MolProbity (45) within PHENIX. The resolution of the model was estimated as 3.0 Å, according to the model vs map FSC = 0.5 criterion (Supplementary Figure 3G).

### Atomic model analysis and figure making

Figures of atomic models and cryo-EM maps were made using UCSF ChimeraX. Virtual amino acid mutation was carried out using the ‘swapaa’ function in ChimeraX, which picks the best rotamer based on clash score, hydrogen bonding and prevalence according to the Dunbrack library (46,47). Cryo-EM consensus and multibody refinement maps used for model building are available in the EMDB (EMD-13191), along with half-maps and masks. The atomic model is available in the PDB (7P48).

### Sal(B) mutagenesis

DNA corresponding to *sal* genes containing mutations of interest was obtained from Genewiz. Cloning of these DNA fragments in *S. aureus* RN4220 using plasmid pRMC2, and susceptibility testing of the resulting constructs, was performed as described above.

## RESULTS

### *sal*-type determinants as a common source of PLM resistance in non-aureus staphylococci

**Figure 1.**
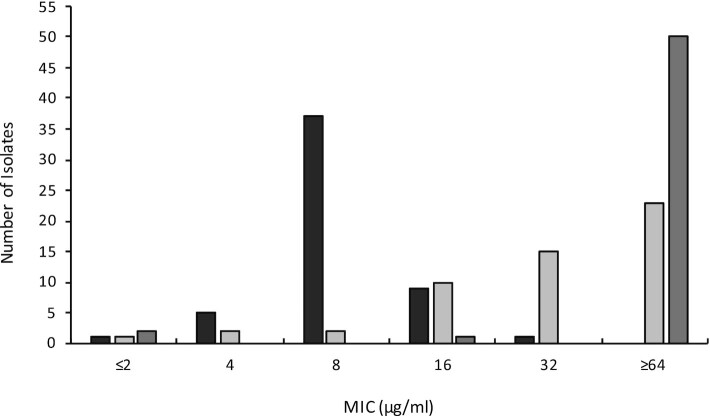
Pleuromutilin susceptibility profiles of the 53 staphylococcal isolates identified in this study that exhibit reduced susceptibility to retapamulin. Retapamulin is shown in black, tiamulin in dark grey, and lefamulin in light grey.

The emphasis in studies on PLM resistance in staphylococci has to date been on *S. aureus*; the starting point for the present study was to explore the nature of PLM resistance in other members of this genus, which are collectively an important cause of infection in humans and animals. Of a collection of 363 non-aureus staphylococci, 53 (∼15%) were found to be capable of growing on agar containing the PLM retapamulin at a concentration corresponding to the proposed epidemiological cut-off (ECOFF) value for resistance in staphylococci (2 μg/ml) (20). The majority of these resistant isolates originated from veterinary sources (*n* = 41), whilst the remainder were isolated from humans. Susceptibility testing established that the minimum inhibitory concentration (MIC) of retapamulin for these isolates ranged from 2 to 32 μg/ml, with the majority (∼70%) associated with an MIC of 8 μg/ml (Figure 1). To assess whether these isolates also exhibited reduced susceptibility to other members of the PLM class, we performed susceptibility testing with tiamulin and lefamulin (Figure 1). On the basis of suggested breakpoint/ ECOFF values for tiamulin (2 μg/ml) (48) and lefamulin (0.25 μg/ml) (6), ∼96% of the retapamulin-resistant isolates exhibited cross-resistance to both of these agents.

The genetic basis for resistance in these isolates was investigated by PCR amplification using oligonucleotide primers designed to amplify known PLM resistance determinants. Six isolates yielded a PCR product with primers directed to *vga(A)*, and subsequent DNA sequencing of these amplicons revealed that they corresponded to *vga(A)*_LC_ or closely-related variants thereof (*data not shown*). For the remaining 47 isolates, a PCR amplicon was generated with primers targeted to *sal(A)*. Sanger sequencing of these amplicons confirmed that they corresponded to the *sal(A)* gene and closely-related variants (>98% identity in the encoded protein) in 28 of the 47 PCR-positive isolates, all of which were subsequently determined to be *S. sciuri*. Of the remaining 19 isolates, three were *Staphylococcus lentus* strains that all harboured a near-identical *sal*-type gene exhibiting considerable polymorphism relative to *sal(A)*; across the length of the ∼325 bp amplicon generated, the sequence showed only ∼68% predicted amino acid identity to Sal(A). To obtain the full sequence of this *sal* gene, a representative isolate (*S. lentus* B3) was subjected to WGS (sequence deposited under NCBI Accession number JAHWBZ000000000). The complete *sal* determinant encodes a protein (MBW0770001) that exhibits 68% identity to Sal(A). Based on the precedent of 80% amino acid identity to represent the dividing line between a known resistance determinant and a novel one (49), we designated this resistance protein Sal(B) (Supplementary Figure S1). Of the remaining 16 isolates that yielded a PCR product with the *sal(A)* primers—all of which were determined to be *Staphylococcus fleuretti—*sequencing of the amplicon revealed a gene encoding a Sal protein distinct from both Sal(A) and Sal(B). The full sequence was obtained by WGS of a representative isolate (*S. fleurettii* A6; Genbank Accession JAAQPD000000000). The encoded protein (MBW0764195) exhibits 71% and 68% identity to Sal(A) and Sal(B), respectively, and was designated Sal(C) (Supplementary Figure S1).

To confirm that these novel *sal*-type genes are capable of conferring the PLM resistance phenotype detected in the strains that harbour them, regulated expression constructs carrying these determinants were introduced into the PLM-susceptible cloning host, *S. aureus* RN4220. Susceptibility testing of the resulting strains established that *sal(B)* and *sal(C)* conferred substantial reductions in susceptibility to PLMs that were comparable to or greater than those observed for an equivalent construct expressing *sal(A)* (Table 1). In addition to PLM resistance, *sal(A)* is reported to confer resistance to lincosamides and group A streptogramins, but does not impact susceptibility to macrolides or group B streptogramins; this same resistance profile was also observed for *sal(B)* and *sal(C)* (Table 1).

**Table 1.** Antibiotic resistance profile conferred by *sal-*type genes in *S. aureus* RN4220

	MIC (μg/ml)						
	*Pleuromutilins*		*Lincosamides*		*Streptogramin (A)*	*Macrolide*	*Streptogramin (B)*
Construct expressing	Retapamulin	Tiamulin	Lincomycin	Clindamycin	Virginiamycin M1	Erythromycin	Pristinamycin IA
empty vector (control)	0.06	0.25	0.25	0.06	1	0.25	4
*sal(A)*	4	8	8	2	4	0.25	4
*sal(B)*	8	16	8	1	2	0.25	4
*sal(C)*	16	4	4	1	4	0.25	4
*sal(D)*	4	8	1	0.06	2	0.25	4
*sal(E)*	2	2	0.25	0.06	1	0.25	4

### *In silico* detection of further novel *sal*-type determinants

The finding that several distinct *sal* determinants confer PLM resistance among the strains examined here led us to investigate whether further, novel *sal*-type PLM resistance genes might also exist within this genus. BLAST searching of the deposited genome sequence data for non-aureus staphylococci identified a range of additional homologues, all of which have amino acid sequence identities to Sal(A) of <50%. Five diverse representatives were selected from these homologues for further analysis; WP_082039181.1 from *Staphylococcus gallinarum* (45% identity to Sal(A)), WP_107546009.1 from *Staphylococcus xylosus* (41% identity to Sal(A)), WP_096809342.1 from *Staphylococcus nepalensis* (43% aa identity to Sal(A)), WP_107510893.1 from *Staphylococcus equorum* (42% aa identity to Sal(A)), and WP_081327038.1 from *Staphylococcus saprophyticus* (40% aa identity to Sal(A)). The selected genes were obtained by synthesis and introduced into *S. aureus* RN4220 for susceptibility testing as described above. The *sal-*type gene from *S. gallinarum* conferred a comparable reduction in PLM susceptibility to that associated with *sal(A)*, and was given the designation *sal(D)*. Intriguingly, Sal(D) was less effective in reducing susceptibility to lincosamides compared with Sal(A)-(C); this protein mediated only a 4-fold decrease in lincomycin susceptibility (4–8-fold lower than that seen for the other *sal* genes), and had no effect on clindamycin susceptibility (Table 1). The gene from *S. nepalensis* also conferred a reduction in PLM susceptibility, but to a lesser degree than generally seen for the other *sal* genes tested, and had no apparent effect on susceptibility to lincosamides or group A streptogramins. This determinant was given the designation *sal(E)* (Table 1). None of the other three *sal*-type genes examined caused a change in susceptibility to the antibiotics tested.

Collectively, we have therefore distinguished five Sal-type ABC-F proteins showing considerable sequence diversity (sequence alignment in Supplementary Figure S1) that all mediate PLM resistance, but which vary in the level of protection they offer against PLMs, and in their ability to mediate resistance to other antibiotic classes. Furthermore, it appears that some *sal*-type genes do not mediate antibiotic resistance.

### Phylogenetic analysis and genetic environment of *sal-*type determinants in staphylococci

**Figure 2.**
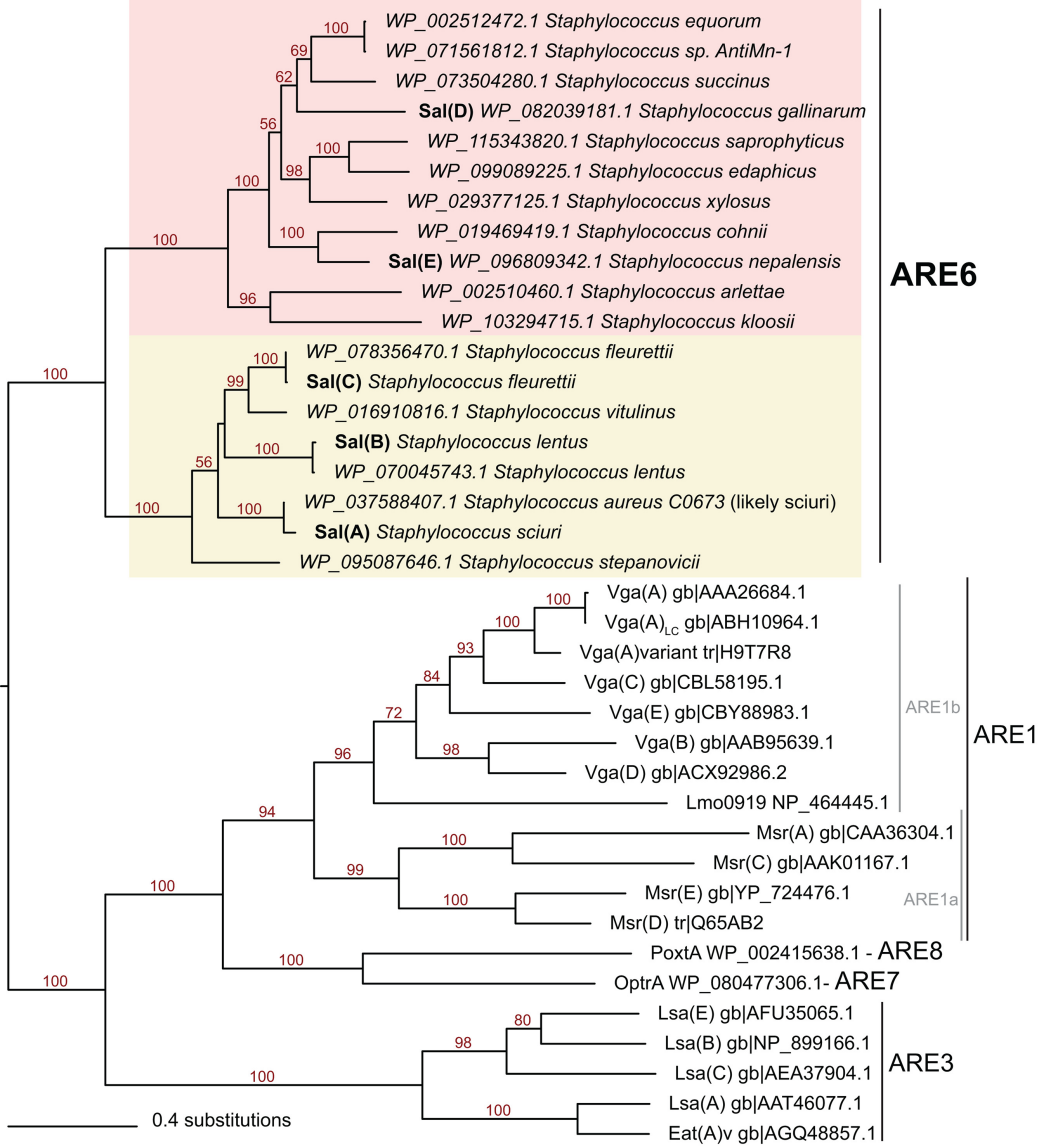
Diversity of Sal-type proteins. An unrooted, maximum likelihood phylogeny of novel and variant Sal-type proteins and other selected ABC-F proteins involved in antibiotic resistance shows a bipartite structure of the Sal-type (ARE6) subfamily, with Sal(A)–(C) and Sal(D)–(E) falling within two respective, fully-supported clades. Branch lengths are proportional to the number of amino acid substitutions as per the lower left scale bar. Numbers on branches indicate percentage bootstrap support values.

According to a recently established classification scheme for ABC-F proteins, Sal(A) resides within a subfamily designated ARE6 (18). Phylogenetic analysis shows that this subfamily comprises a distinct group with a bipartite structure (Figure 2), and confirms that all *sal*-type determinants identified in this study—including those with only low sequence identity to Sal(A)—are true members of the subfamily. Reflecting the observation above that Sal(D)-(E) do not exhibit the classical Sal(A) antibiotic resistance profile, these two proteins cluster in a clade distinct from Sal(A)–(C) (Figure 2).

**Figure 3.**
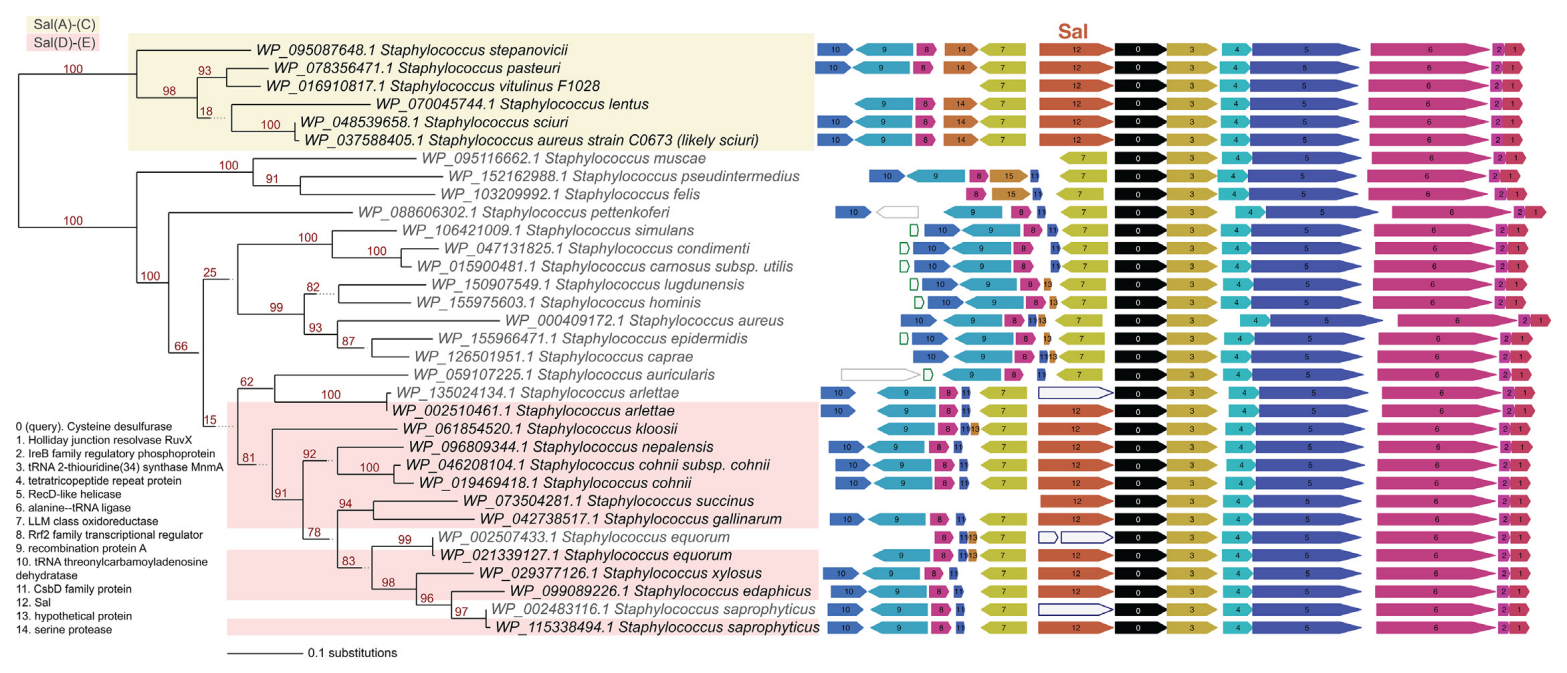
*sal* genes reside in a well-conserved genetic context that is present even in species lacking *sal*. Query proteins for the FlaGs neighbourhoods and phylogenetic analysis are cysteine desulfurases. Colours and numbers indicate genes encoding homologous proteins, with the legend for functional annotations inset on the left. The Sal-type protein coding genes are shown in orange. Taxa highlighting indicates whether the Sal-type protein belongs to Sal(A)–(C) (yellow) or Sal(D)–(E) (pink). Green-outlined grey genes are non-coding genes, and blue-outlined genes show pseudogenised *sal* genes. Numbers on branches indicate percentage bootstrap support.

As Sal proteins are not universally encoded in staphylococcal genomes, we examined whether this might be indicative of mobility by comparing the genomic regions where these are encoded. Genomic context is well conserved around *sal* genes, with only minor gene neighbourhood differences between *sal(A)-(C)-*type and *sal(D)-(E)* type (Figure 3), as has been observed previously for *sal(A)* (16). To rule out the possibility that a larger region of the genome containing *sal* is being horizontally transferred (e.g. on a transposon), we retrieved protein homologues encoded by the downstream gene (cysteine desulfurase) and ran neighbourhood analysis on these sequences. Cysteine desulfurase is a near-universal protein encoded within the genomes of staphylococci, the gene for which resides in the same well-conserved gene cluster with or without *sal* as the upstream gene (Figure 3) (17). This implies that *sal* genes are not mobilising, supporting the suggestion made previously regarding *sal(A)* that these genes are intrinsic to the species in which they are found (17). Furthermore, the finding that Sal phylogeny (Figure 2) is congruent with species phylogeny (Figure 3) supports the idea that *sal* genes are not routinely spread among staphylococci by horizontal gene transfer. Rather, it implies that *sal* genes are ancestral to the genus, and that the discontinuous distribution of *sal-*type genes across the staphylococci is due to gene loss. In fact, this gene loss appears to have happened—and still be happening—independently in multiple lineages; in some strains that carry *sal*, this gene has become pseudogenised (Figure 3).

### Structural and functional insights into Sal-type proteins

To begin to explore the molecular detail of Sal-type antibiotic resistance and determine whether Sal-type proteins mediate resistance in a manner analogous to other ARE ABC-F proteins (i.e. by ribosomal protection), we first examined whether we could detect interaction between a representative Sal-type protein (Sal(B)) and the staphylococcal ribosome. It has been shown for other ABC-F proteins that, when defective in NTPase activity, they are unable to dissociate from the ribosome once bound (12,50–52). On that basis, we engineered an NTPase-deficient (EQ_2_) mutant of Sal(B), which was expressed in *S. aureus*; affinity purification of this FLAG-tagged Sal(B) from cell lysates successfully pulled down 70S ribosomes, as determined by negative stain EM (*data not shown*).

**Figure 4.**
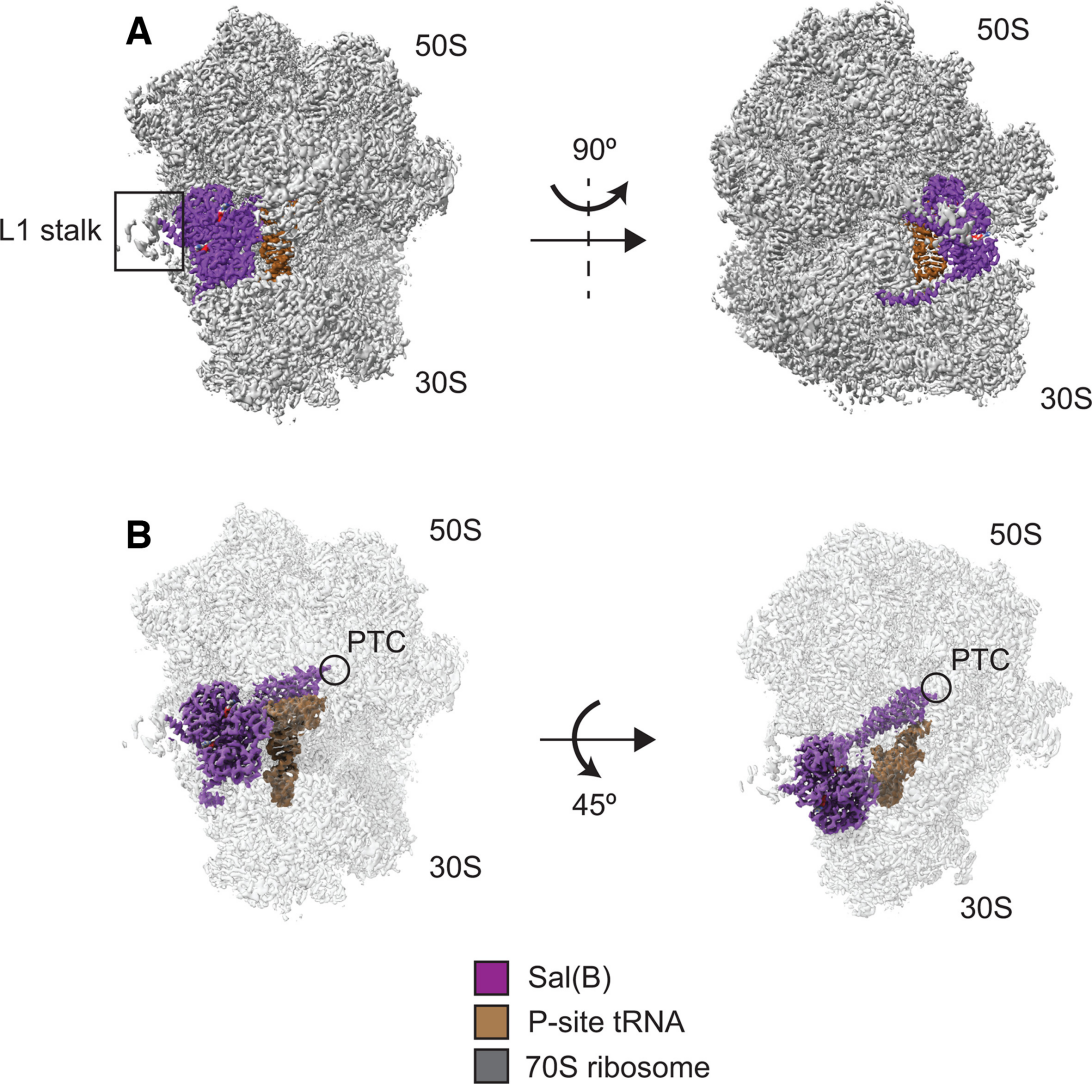
Structure of the Sal(B)•ribosome complex. Sal(B) (purple) binds to the E-site of the ribosome (grey), with the interdomain linker of the former contacting and distorting the P-site tRNA (brown). ATP molecules coloured by atom. (**A**) Left; front view showing the NBDs of Sal(B) bound to the E site, in close proximity to the L1 stalk. Right; side view showing the C-terminal tail of Sal(B) wrapping around the 30S subunit. (**B**) Left; front view with transparent ribosome density to show the interdomain linker of Sal(B) reaching towards the peptidyl transferase centre (PTC) of the 50S ribosomal subunit, where LS_A_P antibiotics bind. Right; top view showing the interaction of the interdomain linker of Sal(B) with the P-site tRNA.

The structure of the resulting Sal(B)•ribosome complex was subsequently solved by cryo-EM to 2.9 Å, and reveals a globular density bound to the *S. aureus* ribosome with a protrusion of density extending towards the P-site tRNA (Figure 4). We ascribed this additional density to Sal(B). The local resolution for this Sal(B) density ranged from 2.6 to 4.6 Å, and for the P-site tRNA from 2.8 to 3.4 Å (Supplementary Figure S4). This allowed an unambiguous atomic model to be calculated for the entire region, with the exception of residues 80–109 in Sal(B) that interact with the L1 stalk of the ribosome. Map and model details and validation statistics are found in Table 2.

**Table 2.** Data collection parameters, image processing, map and model details, and model validation statistics

**Data collection parameters**			
Microscope	Thermo Fisher Titan Krios	**Image processing and map details**	
Detector	Gatan Bioquantum-K2	Micrographs (no.)	847
Detector mode	Counting	Initial particles (no.)	99 615
Voltage	300 kV	Final particles (no.)	59 889
Magnification	130 000 X (nominal)	**Map and model resolutions**	
Electron dose	60 e^–^/Å^2^	Consensus map	2.9 Å (FSC = 0.143)
Exposure time	8.0 s	50S map	2.8 Å (FSC = 0.143)
Dose per frame	1.20 e^–^/Å^2^	30S body map	3.0 Å (FSC = 0.143)
Defocus range	–0.8 to –2.6 μm	30S head map	3.0 Å (FSC = 0.143)
Object sampling	1.07 Å/pixel	Model (fit to consensus map)	3.0 Å (FSC = 0.5)
**Model details and validation statistics**			
**Model composition**		**R.m.s. deviations** ^a^	
Non-hydrogen atoms^a^	138 824	Bond lengths (Å)	0.020
Protein residues^b^	5925	Bond angles (°)	1.47
Nucleic acid residues^b^	4329	**Protein geometry validation** ^a^	
Metal ions^a^	3 Zn, 2 Mg	Rotamer outliers (%)	0.58
Ligand^a^	2 ATP	Ramachandran outliers (%)	0.38
**General validation** ^a^	Ramachandran favoured (%)	86.11	
CC (model to map fit)^c^	0.77	**RNA geometry validation** ^b^	
Clashscore	12.33	Sugar pucker outliers (%)	1.18
MolProbity score	2.24	Backbone conformation outliers (%)	29.96

^a^Obtained from Phenix refine log and phenix molprobity.

^b^Obtained from MolProbity web server.

^c^CC = correlation coefficient, measure of fit into consensus map.

**Figure 5.**
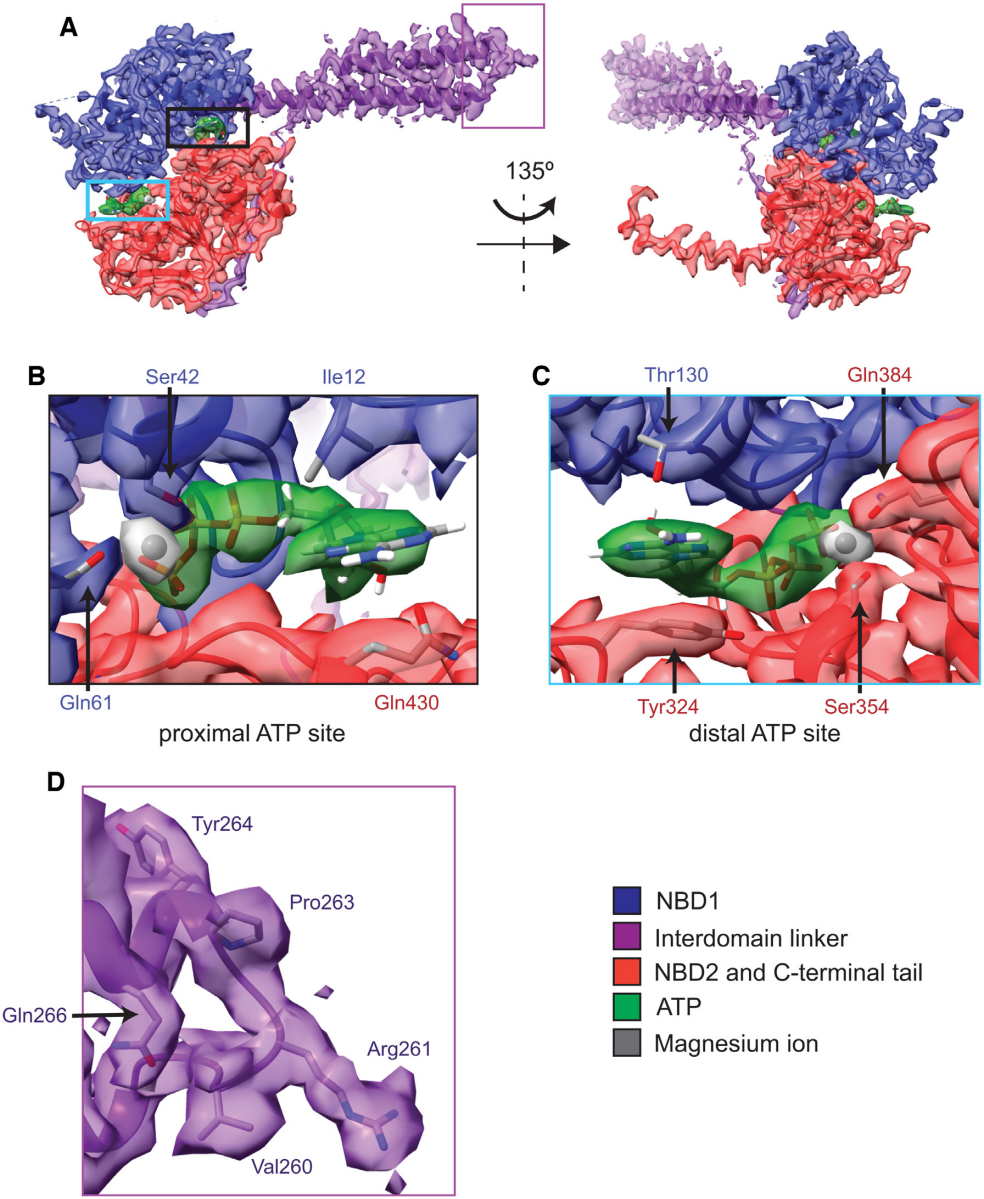
Structure of Sal(B), focussing on ATP-binding sites and the interdomain linker loop. (**A**) Atomic model and cryo-EM density of Sal(B), showing NBD1 (blue), the interdomain linker (purple), NBD2 and the C-terminal tail (red), two sandwiched ATP molecules (green) and magnesium ions that coordinate the β- and γ-phosphates of ATP (grey). Left, front view; right, back view. (**B**) Proximal ATP binding site showing atomic models of the ATP molecule, magnesium ion and selected Sal(B) protein side chains involved in ATP binding. (**C**) Distal ATP binding site showing atomic models of the ATP molecule, magnesium ion and selected Sal(B) protein side chains involved in ATP binding. (**D**) The loop between the two helices of the interdomain linker. The density of this region is well resolved, allowing for unambiguous visualisation of side chains.

Sal(B) comprises an N-terminal nucleotide-binding domain (NBD) (NBD1: Figure 5, in blue) and a second, C-terminal NBD2 (Figure 5; in red), which together bind to the E-site of the ribosome between the L1 stalk and P-site tRNA, in a similar way to other ARE ABC-F proteins (12,50,53). The two NBDs are joined by an interdomain linker (in purple), formed from two alpha helices joined by an interhelix loop; this region of ABC-F proteins is also known as the P-site tRNA Interaction Motif (PtIM) (51,52) or—in the specific case of the ARE ABC­F proteins—the antibiotic resistance domain (ARD) (50). The interdomain linker of Sal(B) extends from the NBDs towards the PTC, the catalytic heart of the 50S ribosomal subunit and the site targeted by lincosamide, group A streptogramins and PLM (LS_A_P) antibiotics (Figure 4). While the domain structure of Sal(B) is similar to that of other ABC-F proteins, there are some localised structural differences, most notably in the interhelix loop (Supplementary Figure S5). Sal(B) has a C-terminal extension that contacts uS7 and uS11 as it wraps around the 30S subunit towards the mRNA exit channel, with residues Asp533, Asn536 and Lys537 closest to the duplex between the mRNA and 16S rRNA in this channel. However, these residues appear to be >7 Å away from the duplex, making an interaction unlikely, and suggesting that the C-terminal extension of Sal(B) is not involved in mRNA recognition (Supplementary Figure S6A, B) (note that this distance is approximate as the density is too weak to model the side chains of Sal(B) or the mRNA:16S rRNA duplex with high confidence (Supplementary Figure S6A, B)). This extension is positioned similarly to the interhelix loop of the C-terminal extension of VmlR (50) (Supplementary Figure S6C).

Two ATP molecules are sandwiched between NBD1 and NBD2 of Sal(B) (green in Figure 5), one proximal to the interdomain linker and the ribosome, and one distal. In each site, the ATP is bound between the Walker A P-loop motif (residues 34–41 of NBD1 at proximal site; 346–353 of NBD2 at distal site) and Walker B β-strand motif (residues 151–155 of NBD1 at proximal site; 451–455 of NBD2 at distal site) of one NBD and the LS(G)GE Signature Sequence of the other (431–435 of NBD2 at proximal site; residues 131–135 of NBD1 at distal site). A number of other interactions also occur. For example, the adenine ring of the proximal ATP molecule is sandwiched between Ile12 of NBD1 and Gln430 of NBD2, and a magnesium ion coordinates the β- and γ-phosphates of ATP with the sidechains of Ser42 and Gln61 from NBD1. Similarly, the adenine ring of the distal ATP is sandwiched between Thr130 of NBD1 and Tyr324 of NBD2, and a magnesium ion coordinates its β- and γ-phosphates with the sidechains of Ser354 and Gln384 from NBD2. The density is well resolved for both ATP molecules, their coordinated magnesium ions and the surrounding protein residues, as well as for the loop joining the two helices of the interdomain linker (Figure 5).

When Sal(B) binds the ribosome, it distorts the acceptor stem of the P-site tRNA away from the PTC, moving the 3′-CCA end by 22 Å compared with its position in an elongation-competent complex (PDB 6O9J) (54) to allow for the interdomain linker loop of the protein to interact with the PTC (Supplementary Figure S7A). This distortion is near identical to that caused by the binding of most other ARE ABC-F proteins whose structures have been determined in complex with the ribosome (12,50) (Supplementary Figure S7B). By contrast, the ARE ABC-F MsrE causes a stronger distortion throughout all regions of the P-site tRNA, with a movement of 28 Å at the 3′-CCA end (53) (Supplementary Figure S7C). As for other ARE ABC-F proteins (12) bound to the ribosome, the structure observed appears to be an initiation complex; the atomic model of *S. aureus* fMet-tRNA_i_^fMet^ fits well into the P-site cryo-EM density, and the model of an AUG mRNA start codon fits into density at the P-site decoding centre (Supplementary Figure S8).

**Figure 6.**
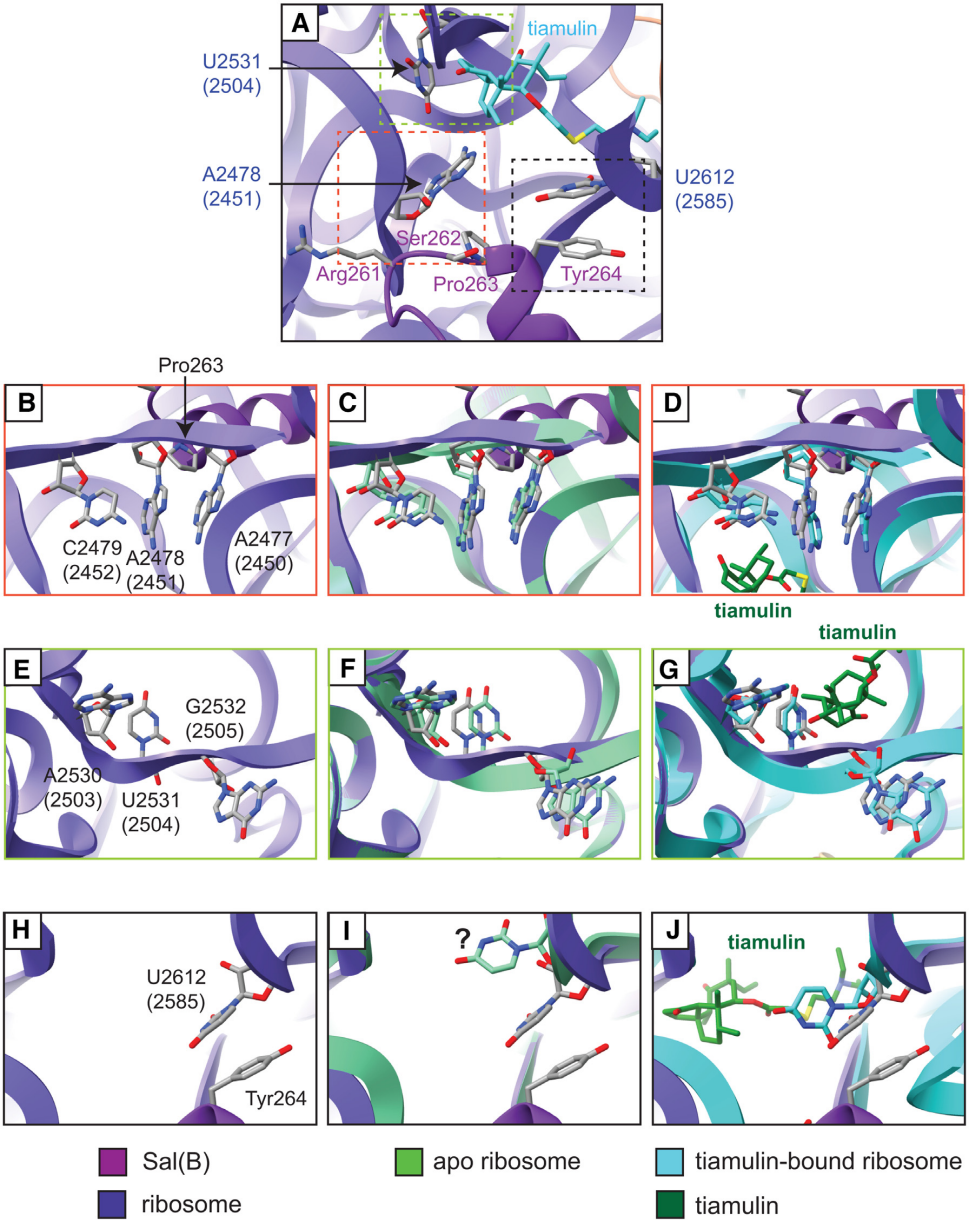
Changes in PTC conformation on binding of Sal(B) or tiamulin to the ribosome. (**A**) Atomic model of the Sal(B)•ribosome complex, zoomed-in on the interdomain linker loop of Sal(B) and ribosome PTC. Sal(B) is shown in purple and 23S rRNA in indigo, with selected residues shown as atomic models. The atomic model of tiamulin from PDB 1XBP is superimposed (light blue) after alignment of the 23S rRNA chains of PDB 1XBP and the Sal(B)•ribosome complex model. No region of the Sal(B) interdomain linker reaches sufficiently close to the drug-binding site to mediate direct displacement of a bound PLM molecule. Dashed coloured boxes outline the regions shown in detail in (B)-(J). (**B, E, H**) Selected regions of the 23S rRNA from the Sal(B)•ribosome complex model. (**C, F, I**) The same regions with a model of the apo *S. aureus* ribosome superimposed. The apo *S. aureus* model was made by refining the relevant 23S rRNA regions from the Sal(B)•ribosome complex model into map EMD-10076. The question mark in part (I) highlights that residue U2612 (2585) of the apo ribosome has poor density and so is likely conformationally flexible. (**D, G, J**) The same regions with a model of the ribosome from *D. radiodurans* in complex with tiamulin superimposed, PDB 1XBP (23S rRNA light blue, tiamulin dark green). For 23S rRNA nucleotides, *E. coli* numbering is shown in parentheses.

The interdomain linker of Sal(B) interacts directly with two 23S rRNA loops at the PTC. First, the backbone of the rRNA loop containing residues A2477, A2478, and C2479 (2450–2452 *E. coli* numbering) interacts with the backbone of Sal(B) residues Arg261 and Ser262, and the ring of Pro263. The closest contacts are made by the ring of Pro263 and the carbonyl oxygen of the backbone of Arg261, which are situated 3.2 and 3.5 Å from the sugar backbone of A2478 (2451), respectively. Second, the base of U2612 (2585) stacks with the aromatic ring of Tyr264 of Sal(B). The aromatic rings of these two residues are situated about 3.4–3.8 Å apart, facilitating a π–π stacking interaction. The sugar oxygen of U2612 (2585) is 3.8 Å from the hydroxy group of Tyr264, which may also allow for weak hydrogen bonding (Supplementary Figure S9). Importantly, no region of the Sal(B) interdomain linker reaches sufficiently close to the drug-binding site to mediate direct displacement of a bound PLM molecule. For example, the distance between Pro263, the closest residue to the antibiotic binding site, and tiamulin (superimposed from PDB 1XBP) is ∼8 Å; too great a distance to allow for any direct interaction, let alone steric displacement (Figure 6A).

Consequently, it seems likely that Sal proteins drive dissociation of PLMs from the ribosome through an allosteric mechanism. There are three regions of the 23S rRNA affected by Sal(B) binding: residues A2477–C2479 (2450–2452), which interact with Sal(B) residues Arg261–Pro263 as discussed (Figure 6B and Supplementary Figure S9C); residues A2530–G2532 (2503–2505), which may interact indirectly with Sal(B) through 23S rRNA residues A2477–C2479 (2450–2452) (Figure 6E); and residue U2612 (U2585), which stacks with Tyr264, as discussed (Figure 6H). Differences in these regions between the apo *S. aureus* ribosome and the Sal(B)•ribosome complex were examined and compared with the published structure of tiamulin bound to the *Deinococcus radiodurans* ribosome (1) to explore how these changes might affect the binding of PTC-targeting antibiotics. First, there is a small shift in the backbone of residues A2477–C2479 (2450–2452) away from the tricyclic core of tiamulin on Sal(B) binding (Figure 6C,D and Supplementary Figure S10A–C), which presumably occurs due to the proximity of Sal(B) residues Arg261–Ser262 and the ring of Pro263. This subtly shifts this region of the 23S rRNA away from the tiamulin molecule, likely weakening binding between the two (Figure 6D). Second, on Sal(B) binding, there is a modest shift in 23S rRNA residues A2530–G2532 (2503–2505), which also form part of the tiamulin binding site (Figure 6F, G and Supplementary Figure S10D–F). Finally, on Sal(B) binding, U2612 (2585) is brought close to Sal(B) Tyr264 such that the aromatic rings of the two residues can form a π–π stack. The density for U2612 (2585) is very weak in the apo ribosome map, suggesting that this residue is conformationally flexible when no Sal protein is bound (Figure 6I and Supplementary Figure S10G–I). On tiamulin binding, this residue moves towards the C-14 glycolic acid chain of tiamulin (Figure 6J). Such an interaction with the drug may not be possible when it stacks with Tyr264, potentially leading to weaker drug binding when Sal(B) is bound.

### Structural and mutational analysis of resistance profiles exhibited by Sal variants

The existence of Sal sequence variants that differ considerably in their antibiotic resistance profiles offered us a useful starting point to interrogate structure-function relationships in this protein family. Thus, we mapped sequences corresponding to the five ARE Sal variants (Sal(A)–(E)) and the three non-ARE Sal variants (Sal proteins from *S. xylosus*, *S. equorum*, and *S. saprophyticus*) onto the structure of the Sal(B) interdomain linker loop (Supplementary Figure S11).

Residues Arg261 and Pro263 are conserved in all cases, but there is variation at position 262; it is a polar serine in Sal(A) and Sal(B) (Supplementary Figure S11A), a slightly larger polar asparagine in Sal(C), Sal(D) and Sal(E) (Supplementary Figure S11B–D), and a negatively-charged aspartate in the non-ARE Sal proteins (Supplementary Figure S11E–G). However, it should be noted that the sidechain at position 262 is not sufficiently close to interact with 23S rRNA in the Sal(B)•ribosome complex, regardless of the residue present. Indeed, even the backbone of residue 262 is further away than the backbone of Arg261 and the ring of Pro263, making it unlikely that this residue plays a major role in antibiotic resistance (Supplementary Figure S9). Nevertheless, it is possible that a change at residue 262 might alter the overall conformation of the interdomain linker loop, in turn affecting the interaction of Sal with the 23S rRNA.

The identity of residue 264 also differs across the variants. It is an aromatic tyrosine residue in Sal(A), Sal(B) and Sal(C) (Supplementary Figure S11A,B), allowing the formation of a π-stack with 23S rRNA residue U2612 (2585), pulling it away from the drug-binding pocket. This π–π stacking interaction is not possible in any of the other Sal variants, which might explain why Sal(A)-(C) generally give the largest increases in LS_A_P resistance. In Sal(D), this residue is leucine, and in Sal(E) and the non-ARE Sal variant from *S. xylosus*, it is isoleucine (Supplementary Figure S11C–E); only weaker hydrophobic interactions with U2612 (2585) would be possible for these non-aromatic residues. In the other two non-ARE Sal variants, position 264 is hydrophilic (Ser or Asn), which would abolish any hydrophobic interactions (Supplementary Figure S11F,G). Although hydrogen bonding interactions between this residue and U2612 (2585) might be possible, it would require precise sidechain positioning. It should be noted this rRNA residue only directly interacts with PLMs and group A streptogramins, but not with lincosamides (Supplementary Figure S12). Therefore, it is difficult to see from this structural snapshot how changes in Tyr264 would affect lincosamide resistance.

To further probe the role of Sal residue 264 in mediating antibiotic resistance, mutagenesis was undertaken. We reasoned that if Tyr264 plays a key role in the resistance associated with Sal variants A-C, then introducing this residue in place of asparagine in the non-ARE Sal variant from *S. saprophyticus* should result in a gain of function (i.e. the ability to mediate PLM resistance). Reciprocal, loss-of-function mutagenesis experiments were also performed at this same site in Sal(B), replacing Tyr264 with either leucine, isoleucine, serine or asparagine, with the expectation of bringing the resistance profiles in each case more in line with those of Sal(D) (leucine), Sal(E)/ the Sal protein from *S. xylosus* (isoleucine), the Sal protein from *S. equorum* (serine) and the Sal protein from *S. saprophyticus* (asparagine), respectively. The effect of these mutations on resistance profile is shown in Table 3.

**Table 3.** Antibiotic resistance profile of engineered variants of Sal-type proteins

	MIC (μg/ml)			
Construct expressing	Retapamulin	Tiamulin	Lincomycin	Clindamycin
**empty vector (control)**	0.06	0.25	0.25	0.06
**Sal(B) (wild-type)**	8	16	8	1
**Sal(B)_Y264L_**	4	16	4	0.5
**Sal(B)_Y264I_**	4	16	4	0.5
**Sal(B)_Y264S_**	2	16	4	0.25
**Sal(B)_Y264N_**	2	16	8	1
**Sal (*S. saprophyticus*)**	0.06	0.25	0.25	0.06
**Sal_N264Y_ (*S. saprophyticus*)**	0.06	1	0.25	0.06

Introducing Tyr264 into the non-ARE Sal variant from *S. saprophyticus* did indeed result in gain in function, yielding a 4-fold reduction in tiamulin susceptibility (Table 3). The fact that this substitution transformed a protein that does not mediate any level of phenotypic antibiotic resistance into one that does suggests that Tyr264 plays a role in resistance in Sal(B)—and by extension, Sal(A) and (C)—at least in the case of tiamulin. However, resistance to other LS_A_P drugs was unaffected, indicating that the interaction mediated by Tyr264 is only one factor in resistance. The loss-of-function experiments had mixed effects on the antibiotic profile of Sal(B) (Table 3). In the case of retapamulin, all mutants showed a reduced ability to mediate resistance compared with wild-type Sal(B), though resistance was not abolished. A similar effect was observed in the case of the lincosamides, lincomycin and clindamycin; for several of the mutants some reduction in resistance was observed, though not for Sal(B)_Y264N_. Surprisingly, substitution of Tyr264 in Sal(B) had no effect on tiamulin resistance, underscoring the idea that other residues within this region are important for PLM resistance.

## DISCUSSION

Collectively, our results provide considerable insight into the nature of Sal-type ABC-F proteins and their role in PLM resistance in the staphylococci. From a mechanistic perspective, we have established that they do indeed function as target protection proteins to mediate resistance to PLM and other antibiotic classes; like other ARE ABC-F proteins, they bind into the E site of the 70S ribosome to effect dissociation of bound drug molecules (12,50,53). The molecular detail of how ARE ABC-F proteins in general achieve this appears to vary among family members and even by antibiotic class, but is the result of the interdomain linker mediating either direct steric displacement of the antibiotic or triggering allosteric change in the antibiotic binding site that prompts drug release (12,55). In the case of Sal(B) – and by implication, other Sal proteins – the interdomain linker does not overlap with the PLM binding site, indicating that resistance is mediated through an allosteric mechanism. A similar conclusion has recently been reached for the mechanism of the other two ARE ABC-F families that mediate PLM resistance in the staphylococci, the Vga- and Lsa-type proteins (12). Whilst we have shown here that the nature of the residue at position 264 of Sal proteins has an important role in PLM resistance, it is nonetheless clear from our data that there must be other residues within the interdomain linker that also contribute to the resistance mechanism.

In addition to the canonical Sal protein, Sal(A), we have now distinguished four other Sal proteins (Sal(B–E)) mediating PLM resistance in staphylococci that differ by ∼30% to >55% in amino acid sequence from Sal(A) and each other, and which vary in their ability to protect the ribosome from antibiotics; members of the Sal(A-C) clade exhibit the typical resistance profile associated with Sal(A) (resistance to PLMs, group A streptogramins and lincosamides), whilst the phylogenetically-distinct Sal(D-E) group shows lower or no resistance to group A streptogramins and lincosamides. Despite the fact that multiple representatives of the Sal proteins mediate antibiotic resistance, several lines of evidence underscore the idea that this is not their original, evolved function. These include the observation made here that several such proteins do not mediate resistance to antibiotics, indicating that resistance is not a universal feature of Sal-type proteins. Furthermore, our analysis of phylogenetic and genomic context strongly implies that *sal* is ancestral to the genus *Staphylococcus*, thereby arguing instead for a housekeeping role for the encoded protein.

The uneven distribution of *sal* across the genus is apparently the result of lineage-specific loss; in some lineages, this seems to be a work in progress, with *sal* in the process of becoming pseudogenised. The simplest explanation for this loss is a modest fitness cost associated with maintenance of Sal that serves to drive its counter-selection over time. As a ribosome-binding ABC-F protein that presumably samples the ribosomal PTC to perform its native cellular role, this fitness cost could conceivably result through competition with other translation factors and/or a reduction in overall translational efficiency. It is not apparent at present why the evolutionary pressures favouring retention or loss of *sal* appear to differ across staphylococcal species, or whether decades of PLM and/or streptogramin use in veterinary (and more recently, human) medicine has latterly made any contribution to selecting for maintenance of this gene in particular lineages. It is however clear that, since *sal* will routinely be present in a particular staphylococcal lineage unless and until it becomes lost, Sal-mediated antibiotic resistance is an intrinsic - rather than acquired – mechanism of resistance, and the presence (or otherwise) of *sal-*type resistance would generally be expected to be uniform across a species.

Our results therefore imply that multiple staphylococcal species are intrinsically resistant to PLMs (and in a proportion of cases, group A streptogramins/ lincosamides) as a consequence of harbouring *sal*-type genes. This includes species that are known to cause disease in humans, including *S. sciuri* (56,57) and *S. lentus* (58,59). Fortunately, the most medically-significant pathogen of the genus, and a major clinical target for PLM therapy in humans—*Staphylococcus aureus—*is a species that has lost *sal*. Whilst we identified a single case in GenBank of a *sal*-type gene annotated within a *S. aureus* genome (strain C0603; Figures 2 and 3), this appears to represent a misidentification of a strain of *S. sciuri* (all five ABC-F proteins found in this strain have top Blastp hits to proteins from *S. sciuri*; *data not shown*). However, the well-documented ability of *S. aureus* to recruit antibiotic resistance determinants from non-aureus staphylococci (e.g. *mecA* (60)*, cfr* (61), and *fexA* (62)) means that this species could recapture *sal* in the future, an event that will be under significant selection by an antibiotic class that is now in both veterinary and human use.

## DATA AVAILABILITY

The cryo-EM map of the Sal(B)•ribosome complex and the associated molecular model have been deposited in the Electron Microscopy Data Bank and Protein Data Bank with the accession codes EMD-13191 and PDB-7P48, respectively.

## Supplementary Material

gkac058_Supplemental_File
