# Supplementary material for: Sal-type ABC-F proteins: intrinsic and common mediators of pleuromutilin resistance by target protection in staphylococci
Source: Nucleic Acids Res. 2022 Feb 7;50(4):2128–42. doi: 10.1093/nar/gkac058 (PMC8887462; doi:10.1093/nar/gkac058)
Supplement: gkac058_Supplemental_File [file gkac058_supplemental_file.pdf]

SUPPLEMENTARY ONLINE MATERIALS

Sal-type ABC-F proteins: intrinsic and common mediators of pleuromutilin resistance by target protection in staphylococci

Merianne Mohamad[1], David Nicholson[1], Chayan Kumar Saha[2,3], Vasili Hauryliuk[2,3], Thomas A. Edwards[1], Gemma C. Atkinson[2,3], Neil A. Ranson[1] and Alex J. O'Neill[1*]

[1] Astbury Centre for Structural Molecular Biology and School of Molecular & Cellular Biology, Faculty of Biological Sciences, University of Leeds, Leeds, UK

[2] Department of Molecular Biology, Umeå University, 90187 Umeå, Sweden

[3] Department of Experimental Medical Science, Lund University, 221 00 Lund, Sweden

Designation	Sequence (5′-3′)	Application
vgaA-F	AGTGGTGGTGAAGTAACACGAAA	Detection of *vgaA*
vgaA-R	TCCGAAGGTTCAATACTCAATCG	
lsaE-F	ACAGCGAGTTGTTTCCTGCT	Detection of *lsaE*
lsaE-R	GCTTTTGCAGCCTTATGTCC	
salA-F	GTTGATTCAGCGATGATGGA	Detection of *salA*
salA-R	CCATGTGGTTTGTTGGTTCA	
salB-F	GGTGGT<u>GGTACC</u>TCGGGAGGCAACATTATGC	Cloning of *salB*
salB-R	GGTGGT<u>GAATTC</u>ACCATTTTATAAAATTGACTAGAAGTCTG	
salC-F	GGTGGT<u>GGTACC</u>GGAATAAGGAGGCAACATTATGC	Cloning of *salC*
salC-R	GGTGGT<u>GAATTC</u>GACTAGAACTCTGTTTCTCTATTTCTTTC	

Supplementary Table 1. Oligonucleotide primers for PCR amplification. Engineered restriction sites are underlined.

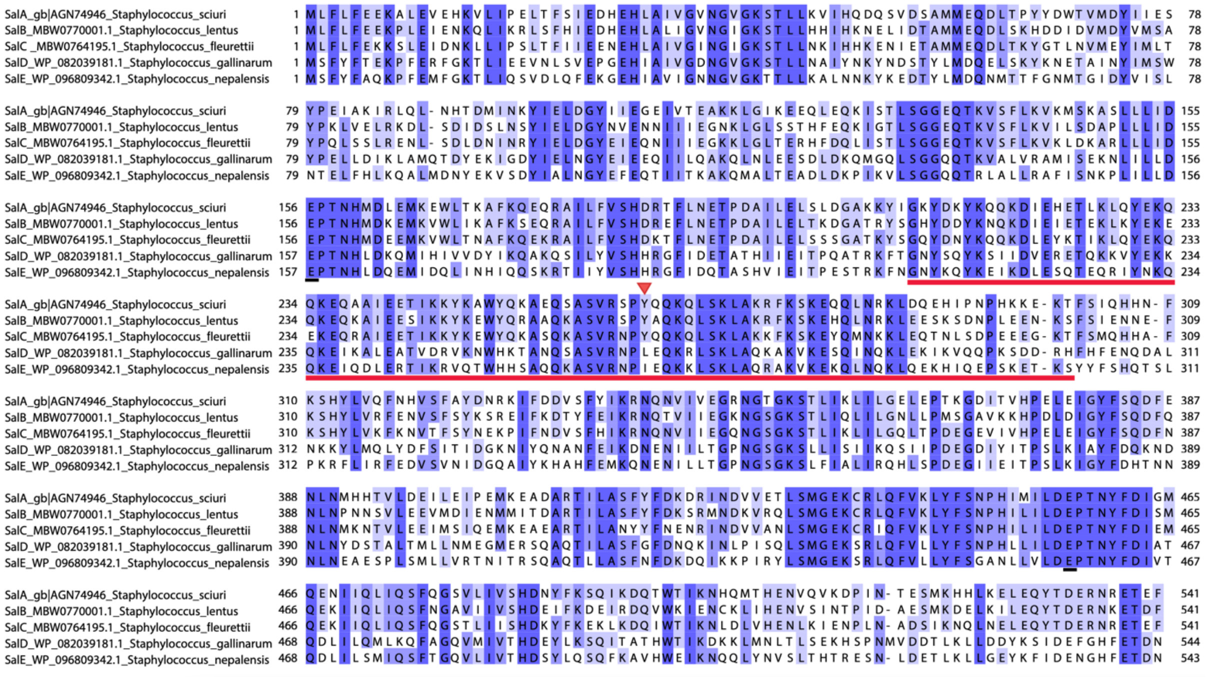

Supplementary Figure 1. Sequence alignment of five distinct Sal-type proteins [Sal(A-E)] that mediate pleuromutilin resistance in staphylococci. Blue highlighting shows the degree of amino acid conservation, with darker colouration indicating greater conservation. The interdomain linker is underlined in red. Key sites at which amino acid substitutions were engineered in this study are indicated; the catalytic glutamate residues within the nucleotide-binding domains that were changed to glutamine in the Sal(B) EQ_2 mutant are underlined in black, whilst the site of the tyrosine residue whose role in antibiotic resistance was explored in mutagenesis experiments is indicated with a red triangle.

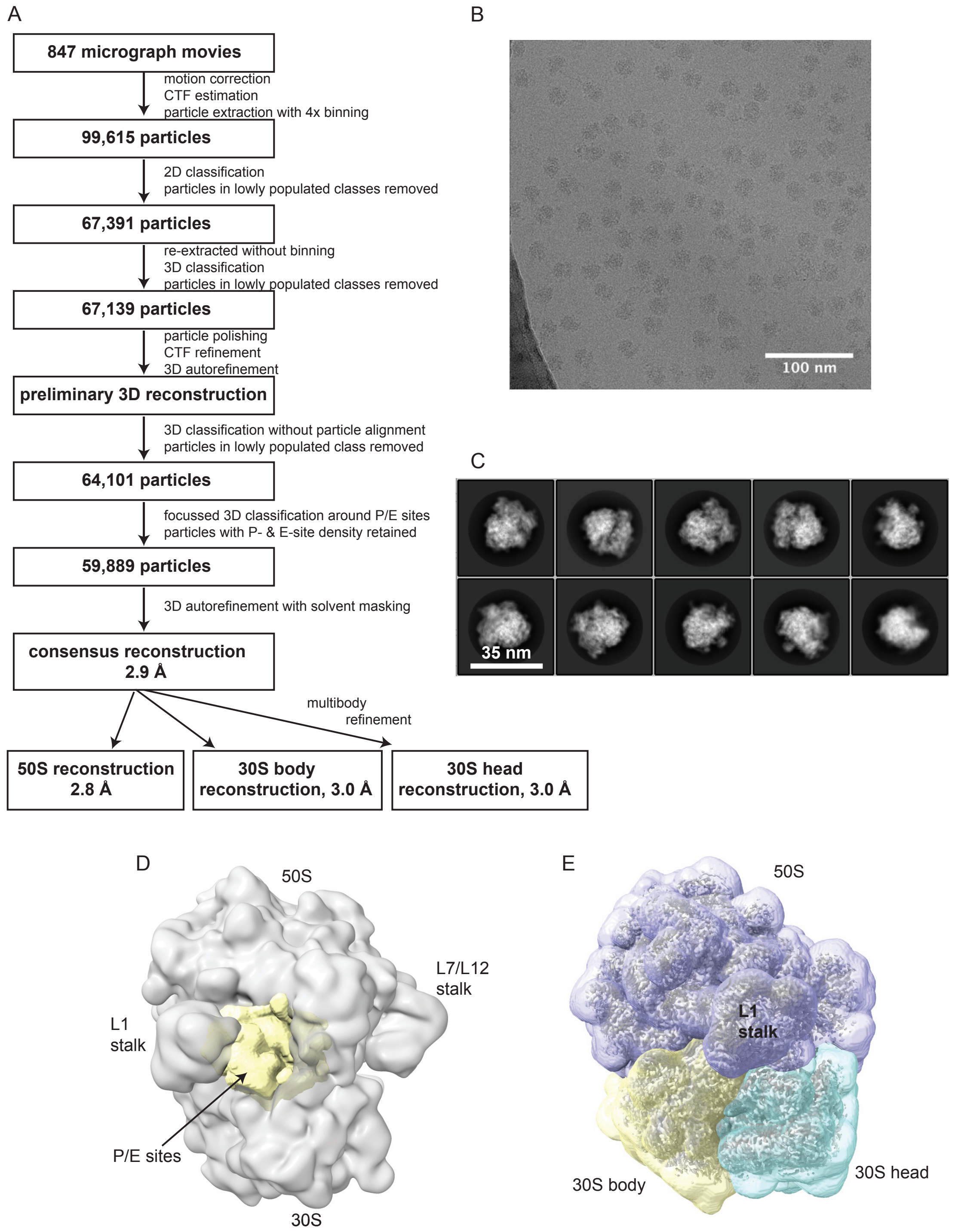

Supplementary Figure 2. Cryo-EM image processing pipeline. **(A)** Image processing flow-chart outlining the steps from micrograph movies to final multibody reconstructions. **(B)** Representative cryo-EM micrograph, scale bar 100 nm. **(C)** Representative 2D class averages, mask diameter 35 nm. **(D)** Mask around the P- and E-sites that was used in the focused classification procedure (yellow), shown around a 3D reconstruction of the complex low-pass filtered to 15 Å (grey). **(E)** 50S mask (purple), 30S body mask (yellow) and 30S head mask (blue) used for multibody refinement, shown around the consensus reconstruction (grey).

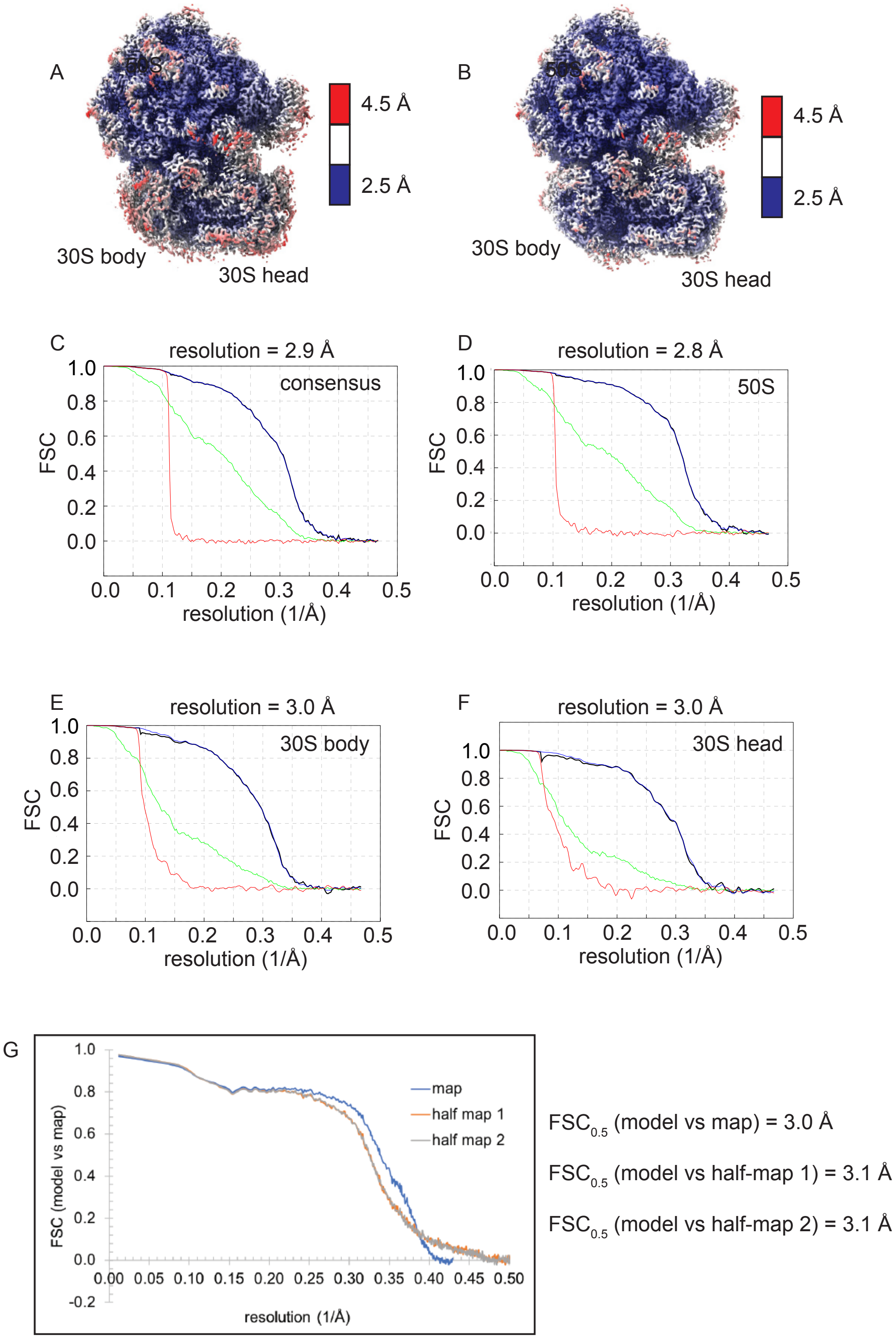

Supplementary Figure 3. Cryo-EM structure determination of the Sal(B)•ribosome complex. (A) Consensus map of the Sal(B)•ribosome complex filtered and coloured by estimated local resolution. **(B)** Superposed maps of the 50S, 30S body and 30S head following multibody refinement, filtered and coloured by estimated local resolution. **(C)** FSC curves between the two half maps as a function of resolution for the consensus reconstruction. The resolution that corresponds to an FSC coefficient of 0.143 is 2.9 Å. **(D)** FSC curves between the two half maps as a function of resolution for the 50S

(*Supplementary Figure 3 legend continued*)

reconstruction. The resolution that corresponds to an FSC coefficient of 0.143 is 2.8 Å. **(E)** FSC curves between the two half maps as a function of resolution for the 30S body reconstruction. The resolution that corresponds to an FSC coefficient of 0.143 is 3.0 Å. **(F)** FSC curves between the two half maps as a function of resolution for the 30S head reconstruction. The resolution that corresponds to an FSC coefficient of 0.143 is 3.0 Å. FSC curves are shown for phase-randomised maps (red), unmasked maps (green), masked maps (blue), and masked maps after correction for mask convolution effects (black). **(G)** FSC curves between the final atomic model and the consensus map (blue) and consensus half maps (grey, orange).

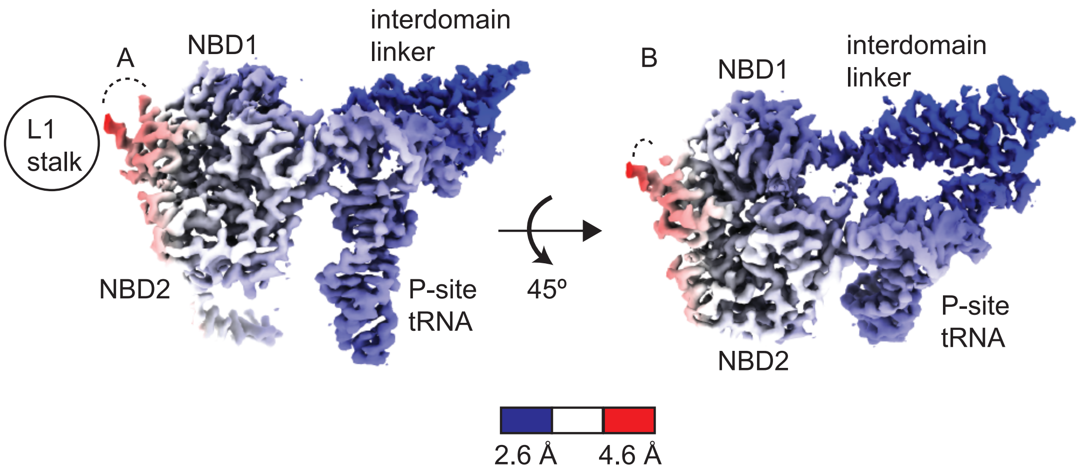

Supplementary Figure 4. Local resolution of the density for Sal(B) and the distorted P-site tRNA. The resolution of the Sal(B) density ranges from 2.6 Å in the interdomain linker to 4.6 Å where NBD1 interacts with the L1 stalk. The resolution of the P-site tRNA density remains fairly consistent, with a small variation from 2.8 Å to 3.4 Å. **(A)** Front view, **(B)** top view. Dashed line represents unbuilt residues 80-109. The relative positioning of the L1 stalk is shown by a black circle.

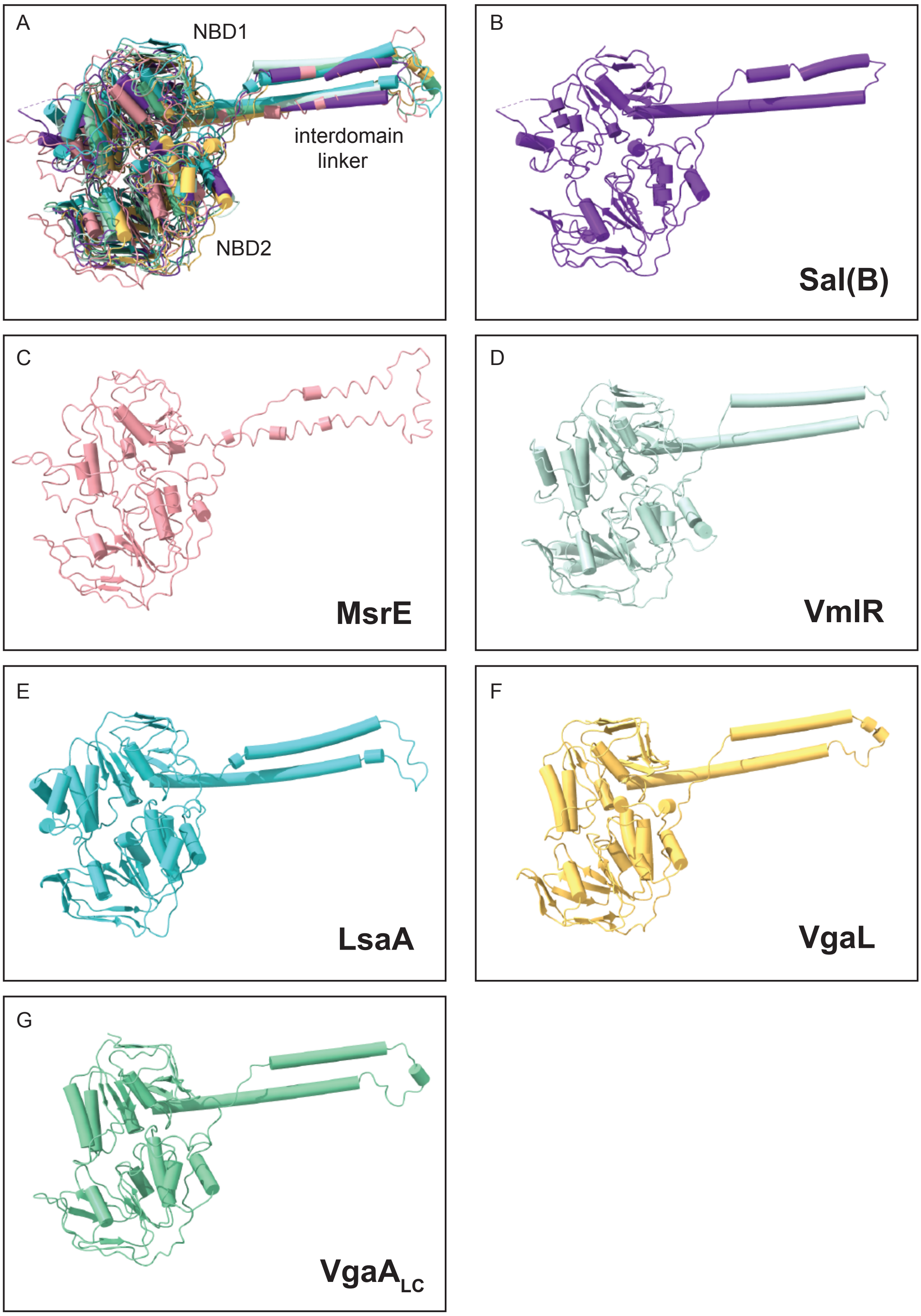

Supplementary Figure 5. Structural comparison of ARE ABC-F proteins. (A) Superposition of six ARE ABC-F proteins in their ribosome-bound form, highlighting the nucleotide-binding domains (NBDs) and interdomain linker. The models are aligned by their 23S rRNA chains (not shown). **(B)** Sal(B) (this study). **(C)** MsrE (PDB 5ZLU), **(D)** VmlR (PDB 6HA8), **(E)** LsaA (PDB 7NHK), **(F)** VgaL (PDB 7NHN), **(G)** VgaA$_{LC}$ (PDB 7NHL).

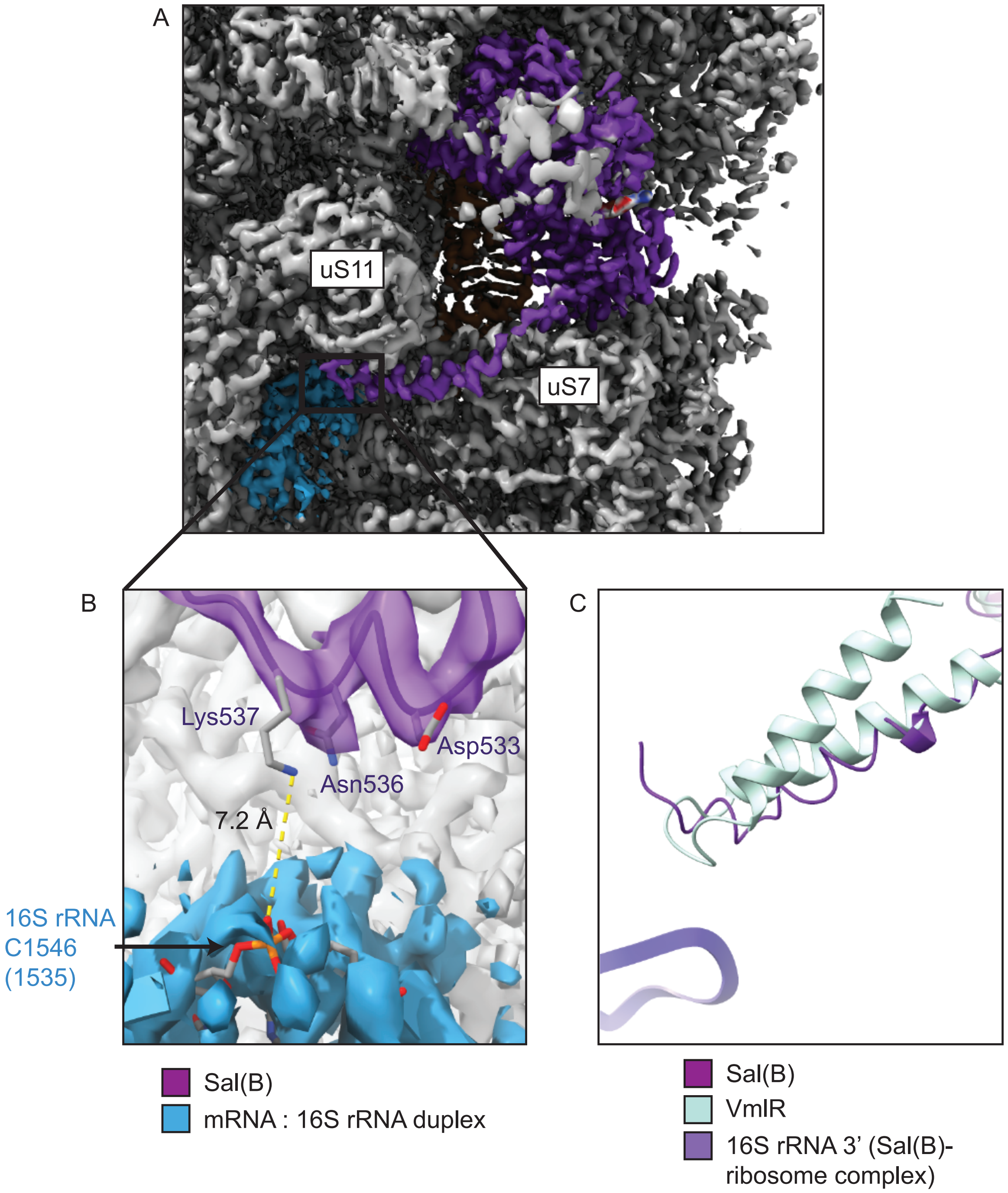

Supplementary Figure 6. The C-terminal extension of Sal(B). (A) Density of Sal(B) (purple) situated in the E site of the ribosome, with C-terminal extension wrapping around the 30S ribosome subunit, making contacts with 30S subunit proteins uS7 and uS11, and reaching towards the mRNA : 16S rRNA duplex in the mRNA exit channel (blue). **(B)** Proximity of the C-terminal extension to the mRNA : 16S rRNA duplex. 16S rRNA residue labelled according to *S. aureus* numbering followed by *E. coli* numbering in parentheses. **(C)** Comparison of the C-terminal extensions of Sal(B) and VmlR (PDB 6HA8), after alignment of the models of the ribosome-protein complexes by their 23S rRNA chains. The nearest part of the 3′ end of 16S rRNA from the Sal(B)•ribosome complex is shown for reference.

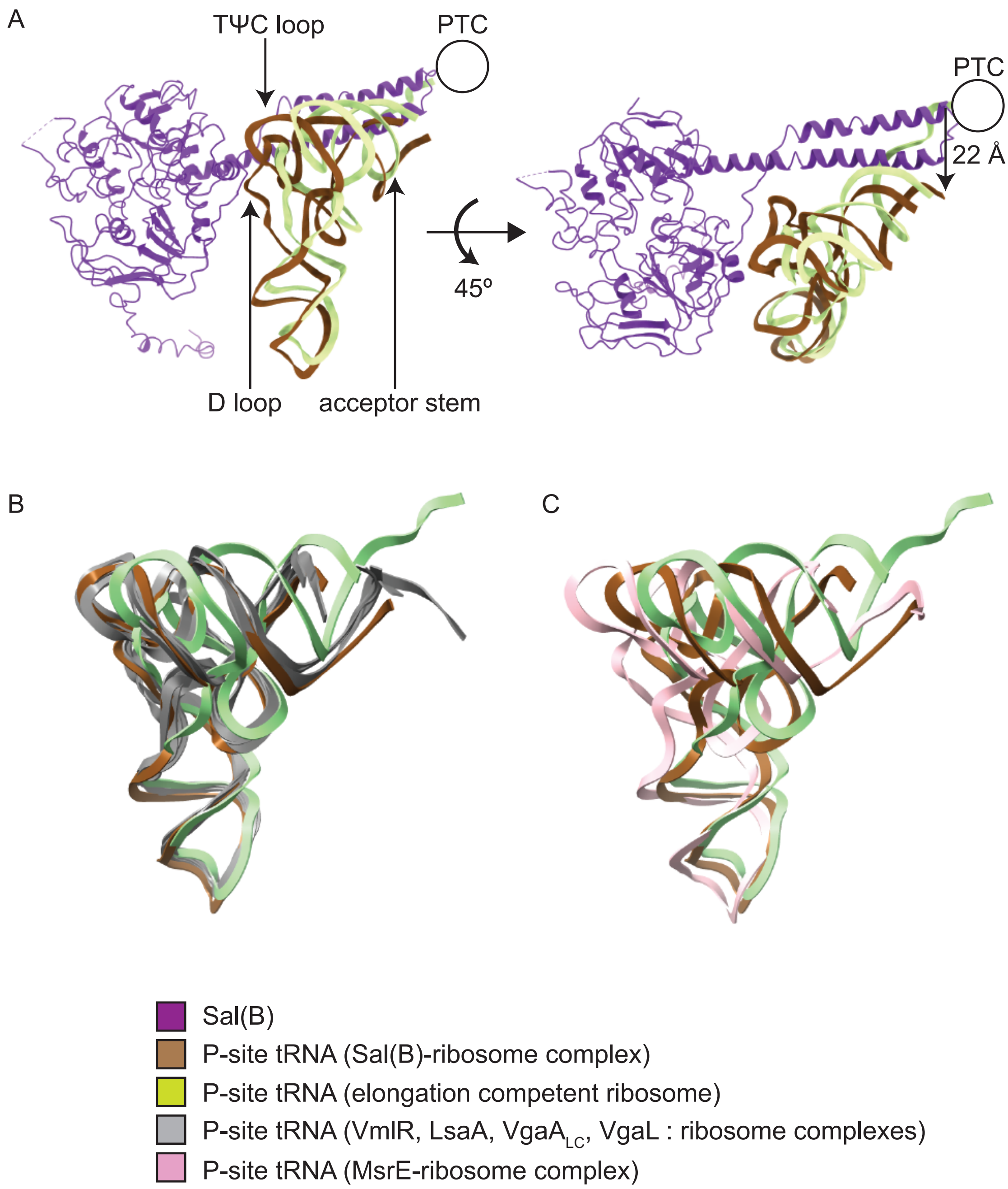

Supplementary Figure 7. Distortion of P-site tRNA on Sal(B) binding. (A) Superposition of P-site tRNA from an elongation-competent ribosome (green; PDB 6O9J) onto the distorted P-site tRNA of the Sal(B)•ribosome complex (brown). The acceptor stem of the elongation-competent P-site tRNA would overlap with the position of the interdomain linker of Sal(B). Therefore, on Sal(B) binding, the acceptor stem of the P-site tRNA is distorted away from the PTC, with the 3'-CCA end moving by 22 Å, as shown. To allow for this movement, the TΨC and D loops of P-site tRNA swing towards NBD2 of Sal(B). The location of the PTC is represented by a black circle. **(B)** Superposition of the P-site tRNA from an elongation-competent ribosome (green; PDB 6O9J) with the distorted P-site tRNAs from the ribosome in complex with Sal(B) (brown), VmlR, LsaA, VgaA$_{LC}$, VgaL (grey; PDBs 6HA8, 7NHK, 7NHL, 7NHN) and **(C)** MsrE (pink, PDB 5ZLU)

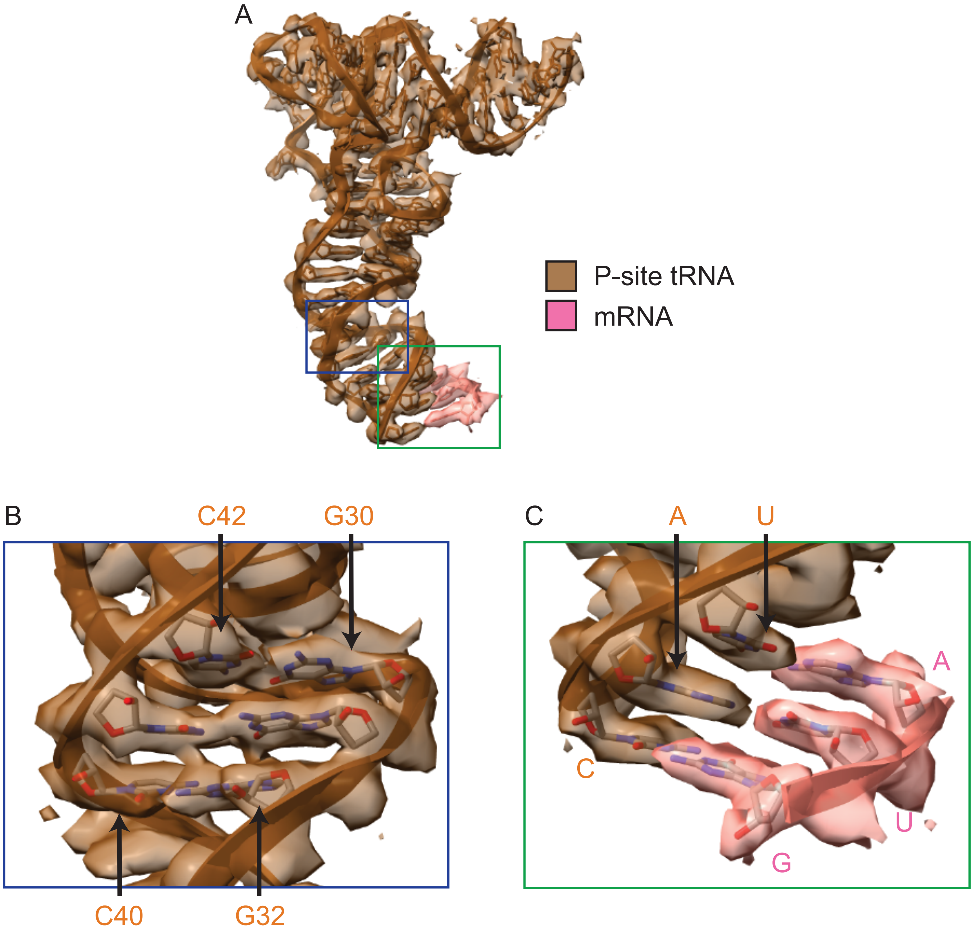

Supplementary Figure 8. Identification of P-site tRNA^{fMet}. (A) A model of *S. aureus* tRNA$_i^{fMet}$ fits well to the P-site tRNA density of the Sal(B)•ribosome complex (brown) and a model of an AUG mRNA start codon fits well into the mRNA density (pink). **(B)** A run of G-C base pairs in the anticodon stem confirms the identification of tRNA$_i^{fMet}$. **(C)** The base-pairing of an AUG mRNA start codon with a CAU anticodon also confirms the identification of tRNA$_i^{fMet}$.

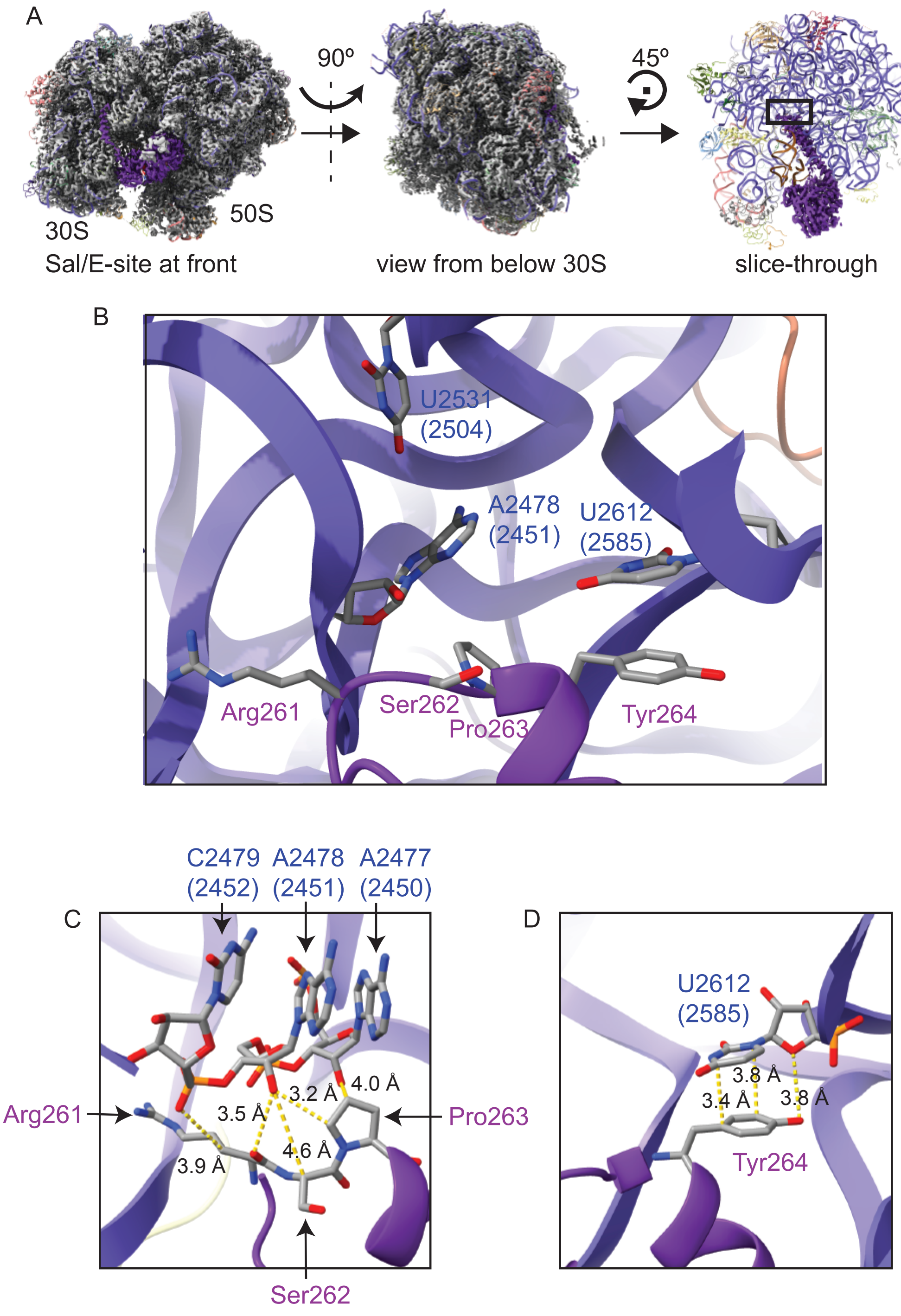

Supplementary Figure 9. Interaction of Sal(B) interdomain linker with the PTC. (A) Sal(B)•ribosome complex with Sal(B) cryo-EM density (purple) and ribosome density (grey), fitted around the atomic model in cartoon form. Left to right: view from E-site, with L1 stalk and Sal(B) at the front; view from below the 30S subunit; in plane rotation and slice through with ribosome density removed to show interaction of the Sal(B) interdomain linker with the ribosome. Zoomed-in view of boxed region shown in part (B). **(B)** Atomic model of the interdomain linker loop of Sal(B) (purple) with key sidechains shown, and atomic model of 23S rRNA (indigo) with key residues shown. **(C)** Atomic model of 23S rRNA residues A2477-C2479 (2450-2452) and Sal(B) residues Arg261-Pro263, with inter-chain distances shown. **(D)** Atomic model of 23S rRNA residue U2612 (2585) and Sal(B) residue Tyr264, with inter-chain distances shown. For 23S rRNA residues, *E. coli* numbering is shown in parentheses.

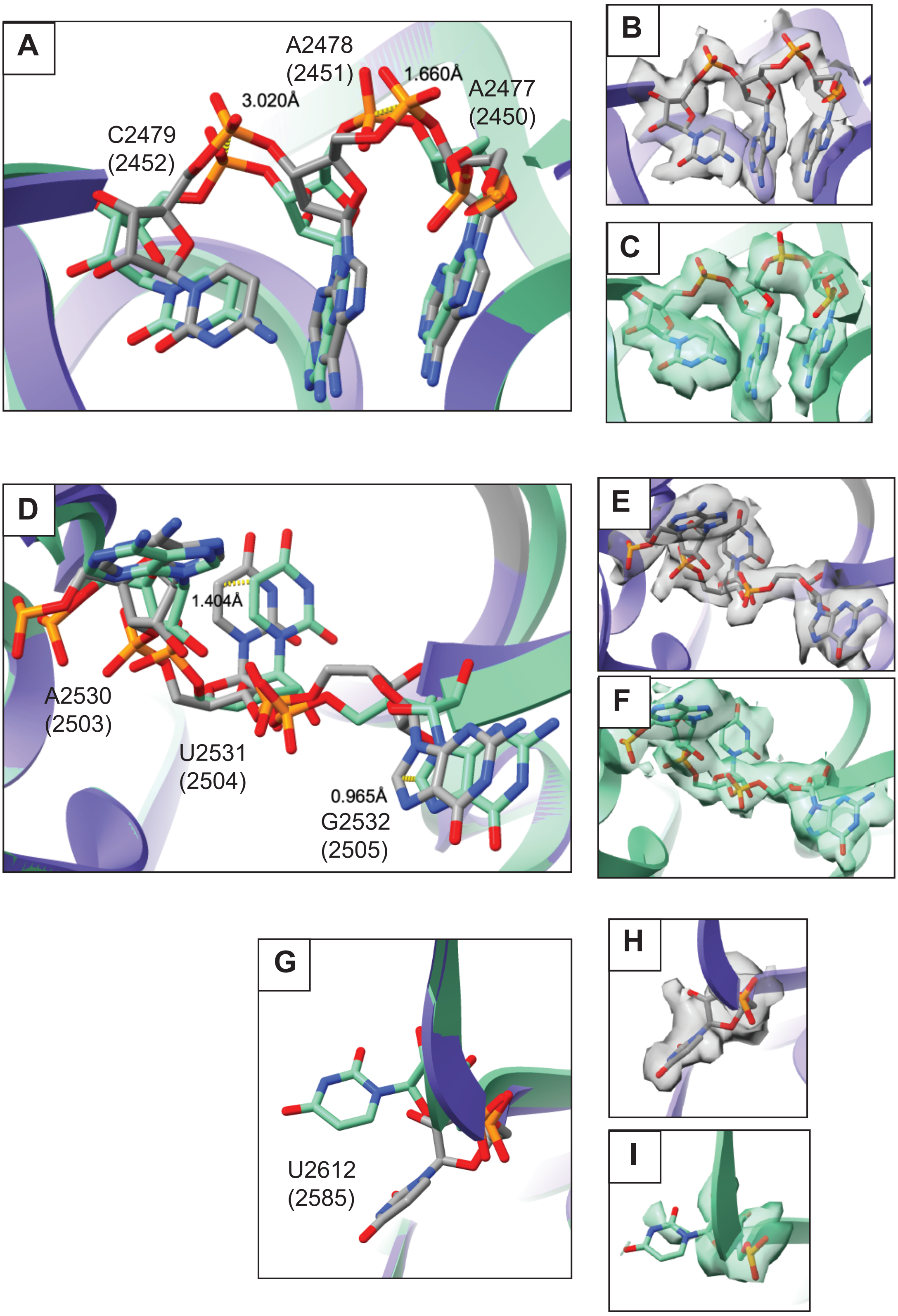

Supplementary Figure 10. Conformational changes in 23S rRNA on binding of Sal(B) to the ribosome. (A) Comparison of the atomic models of the Sal(B)•ribosome complex (purple cartoon, grey stick model) and the apo *S. aureus* ribosome (green cartoon and stick model), focused on 23S rRNA residues 2477-2479 (2450-2452). **(B)** Sal(B)•ribosome complex cryo-EM density of these residues (grey, this

(*Supplementary Figure 10 legend continued*)

study). **(C)** Apo *S. aureus* ribosome cryo-EM density of these residues (green, EMD-10076). **(D)** Comparison of the same atomic models as in (A), focused on 23S rRNA residues 2530-2532 (2503-2505). **(E)** Sal(B)•ribosome complex cryo-EM density of these residues (grey, this study). **(F)** Apo *S. aureus* ribosome cryo-EM density of these residues (green, EMD-10076). **(G)** Comparison of the same atomic models as in (A), focused on 23S rRNA residue U2612 (2585). **(H)** Sal(B)•ribosome complex cryo-EM density of this residue (grey, this study). **(I)** Apo *S. aureus* ribosome cryo-EM density of this residue (green, EMD-10076). The apo *S. aureus* model was made by refining regions of the 23S rRNA of the Sal(B)•ribosome model into map EMD-10076 (green density). For 23S rRNA nucleotides, *E. coli* numbering is shown in parentheses. Distances between select atoms are shown (yellow dashed lines).

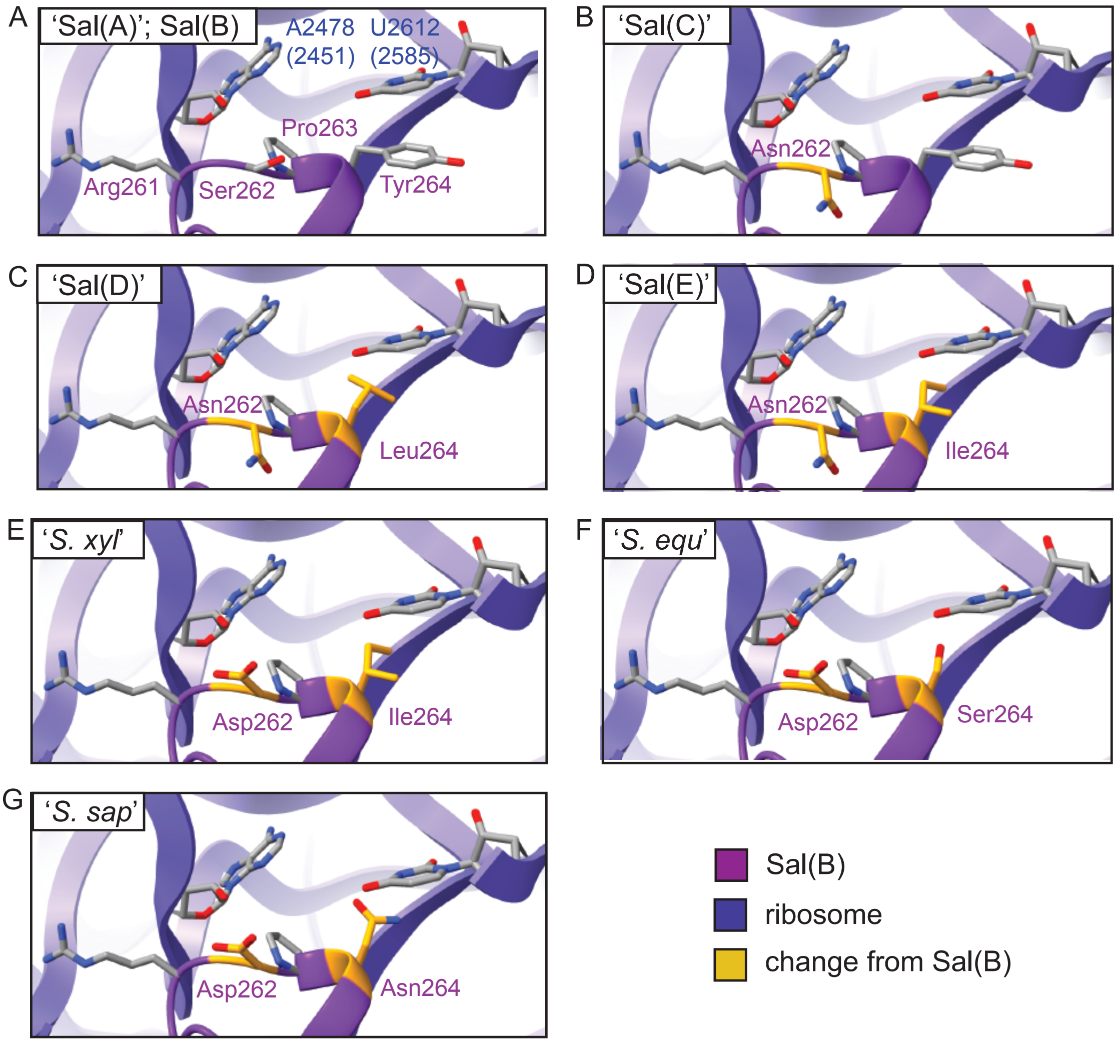

Supplementary Figure 11. Sequences of Sal variants mapped onto the structure of the Sal(B) interdomain linker. (A) Structure of the Sal(B) interdomain linker loop in the Sal(B)•ribosome complex, with residues R261 to 264 shown; these residues are identical in Sal(A) and Sal(B). **(B)** The same structure as in (A) but with Ser262 virtually mutated to Asn to give a representative Sal(C) structure, assuming no change in the overall structure of the linker loop. **(C)** The same structure but with virtual mutations S262N and Y264L to represent Sal(D). **(D)** The same structure but with virtual mutations S262N and Y264I to represent Sal(E). **(E)** The same structure but with virtual mutations S262D and Y264I to represent the Sal protein from *S. xylosus*. **(F)** The same structure but with virtual mutations S262D and Y264S to represent the Sal protein from *S. equorum*. **(G)** The same structure but with virtual mutations S262D and Y264N to represent the Sal protein from *S. saprophyticus*. Residues that differ from Sal(B) are in each case highlighted in gold. For 23S rRNA residues, *E. coli* numbering is shown in parentheses.

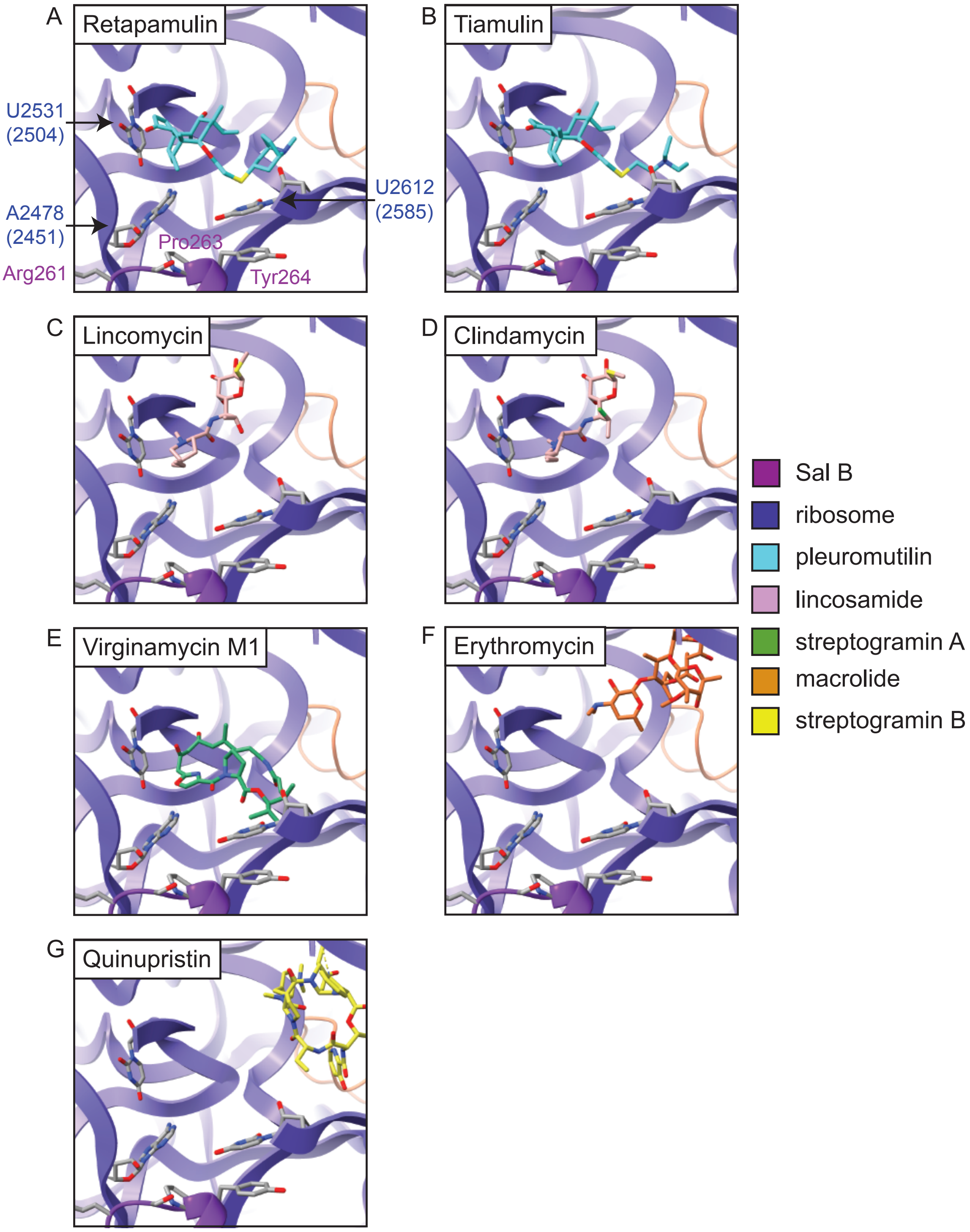

Supplementary Figure 12. Relative positions of Sal(B) and ribosome-targeting antibiotics in the ribosome. Superposition of the atomic models of antibiotics with the Sal(B)•ribosome complex, after alignment of antibiotic-ribosome and Sal(B)•ribosome models by their 23S rRNA chains. **(A)** Retapamulin from PDB 2OGO. **(B)** Tiamulin from 1XBP. **(C)** Lincomycin from 5HKV. **(D)** Clindamycin from 4V7V. **(E)** Virginiamycin M1 from 1YIT. **(F)** Erythromycin from 6S0X. **(G)** Quinupristin from 4U1U.
